# Dynamical–statistical seasonal forecasts of winter and summer precipitation for the Island of Ireland

**DOI:** 10.1002/joc.7557

**Published:** 2022-02-15

**Authors:** Saeed Golian, Conor Murphy, Robert L. Wilby, Tom Matthews, Seán Donegan, Dáire Foran Quinn, Shaun Harrigan

**Affiliations:** ^1^ Irish Climate Analysis and Research Units, Department of Geography Maynooth University Maynooth Ireland; ^2^ Geography and Environment Loughborough University Loughborough UK; ^3^ Department of Geography King's College London London UK; ^4^ Forecast Department European Centre for Medium‐Range Weather Forecasts (ECMWF) Reading UK

**Keywords:** artificial neural network, dynamical models, mean sea level pressure, precipitation, regression, seasonal forecasting

## Abstract

Seasonal precipitation forecasting is highly challenging for the northwest fringes of Europe due to complex dynamical drivers. Hybrid dynamical–statistical approaches offer potential to improve forecast skill. Here, hindcasts of mean sea level pressure (MSLP) from two dynamical systems (GloSea5 and SEAS5) are used to derive two distinct sets of indices for forecasting winter (DJF) and summer (JJA) precipitation over lead‐times of 1–4 months. These indices provide predictors of seasonal precipitation via a multiple linear regression model (MLR) and an artificial neural network (ANN) applied to four Irish rainfall regions and the Island of Ireland. Forecast skill for each model, lead time, and region was evaluated using the correlation coefficient (*r*) and mean absolute error (MAE), benchmarked against (a) climatology, (b) bias corrected precipitation hindcasts from both GloSea5 and SEAS5, and (c) a zero‐order forecast based on rainfall persistence. The MLR and ANN models produced skilful precipitation forecasts with leads of up to 4 months. In all tests, our hybrid method based on MSLP indices outperformed the three benchmarks (i.e., climatology, bias corrected, and persistence). With correlation coefficients ranging between 0.38 and 0.81 in winter, and between 0.24 and 0.78 in summer, the ANN model outperformed MLR in both seasons in most regions and lead‐times. Forecast skill for summer was comparable to that in winter and for some regions/lead times even superior. Our results also show that climatology and persistence performed better than direct use of bias corrected dynamical outputs in most regions and lead‐times in terms of MAE. We conclude that the hybrid dynamical–statistical approach developed here—by leveraging useful information about MSLP from dynamical systems—enables more skilful seasonal precipitation forecasts for Ireland, and possibly other locations in western Europe, in both winter and summer.

## Graphical abstract

In this study, hindcasts of mean sea level pressure (MSLP) from dynamical systems (GloSea5 and SEAS5) are used to derive two distinct sets of indices to forecast winter and summer precipitation. These indices provide predictors of seasonal precipitation via a multiple linear regression model (MLR) and an artificial neural network (ANN) applied to four Irish rainfall regions and the Island of Ireland. Both MLR and ANN models produced skilful precipitation forecasts of up to 4 months lead‐times with our hybrid method based on MSLP indices outperforming direct use of dynamical forecasts, climatology and persistence in every case.

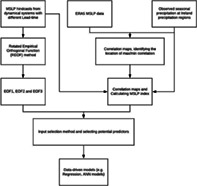

## 1 INTRODUCTION

Seasonal forecasts of climate‐ and water‐related variables are increasingly used, especially within industrial, agricultural, environmental, water, and urban sectors (Agrawala *et al*., 2001; Hewitt *et al*., 2013). Consequently, recent decades have seen considerable advances in the development of monthly and seasonal forecasting systems at global to regional scales (e.g., MacLachlan *et al*., 2015; Yuan *et al*., 2015; Tompkins *et al*., 2017; Emerton *et al*., 2018). There is recognition that seasonal climate variability can be attributed to atmospheric teleconnections, with many studies showing relationships between local climate conditions and large‐scale modes as predictors for skilful seasonal precipitation and streamflow forecasting (e.g., Svensson *et al*., 2015; Mekanik *et al*., 2016; Bell *et al*., 2017; Mariotti *et al*., 2018).

Statistical methods for seasonal forecasting employ techniques include simple linear regression (Ranhao *et al*., 2008; Hall *et al*., 2017; Wang *et al*., 2017) and more sophisticated artificial intelligence (AI) based methods (e.g., da Paz *et al*., 2011; Nourani *et al*., 2019). These transfer functions typically relate climate signals such as sea surface temperatures (SSTs), sea level pressure (SLP) (and/or their derivatives), to precipitation, temperature, or streamflow at target locations (e.g., Ding and Ke, 2013; Mekanik *et al*., 2016; Devi *et al*., 2020). Among climate indices, those based on mean sea level pressure (MSLP), such as the North Atlantic Oscillation (NAO), are known to predict climate conditions over regions of the mid–high latitudes in the Northern Hemisphere, especially western Europe, including the UK and Ireland (Wilby *et al*., 1997; 2004; Wedgbrow *et al*., 2002; 2005; Troccoli, 2010). For example, Murphy and Washington (2001) showed that there is a strong correlation between NAO and precipitation in some parts of the UK and Ireland, particularly in winter. Similarly, Fowler and Kilsby (2002) note the strong connection between the NAO and winter precipitation in Yorkshire, UK. Hurrell and Deser (2010) found that positive phases of the NAO are associated with mild and wet winters in western Europe. West *et al*. (2019) show a spatial–temporal relationship between monthly precipitation and NAO with a clear divide in rainfall patterns across the north/west and south/east regions of the UK during winter months. The NAO can also influence summer precipitation, but signals tend to be weaker compared with winter (Folland *et al*., 2009; Dunstone *et al*., 2018).

The above associations have been incorporated within various statistical approaches to seasonal forecasting of climatological and hydrological variables (e.g., Wilby, 2001; Wedgbrow *et al*., 2002; 2005; Wilby *et al*., 2004; Rodrigues *et al*., 2014; Hall and Hanna, 2018; Lledó *et al*., 2020). Others have indirectly employed these relationships to condition persistence‐based forecasts and ensemble streamflow predictions (e.g., Svensson, 2016; Donegan *et al*., 2021). Furthermore, Murphy *et al*. (2020) demonstrated that monthly and seasonal precipitation in England and Wales and Ireland can be reconstructed from MSLP and climate indices, especially those based on concurrent SLP.

There have also been developments in seasonal forecasts based on numerical weather prediction models that represent the climate system via physical equations to forecast climate evolution several months in advance (e.g., Doblas‐Reyes *et al*., 2006; MacLachlan *et al*., 2015; Johnson *et al*., 2019). Two widely used dynamical forecast systems are the European Centre for Medium‐Range Weather Forecasts (ECMWF) Seasonal Forecasting System (SEAS5) and the UK Met Office's Global Seasonal forecast system version 5 (GloSea5) (MacLachlan *et al*., 2015). Climate signals from SEAS5 and GloSea5 have been used widely by researchers to forecast temperature, precipitation, and wind speed (Baker *et al*., 2018b; Thornton *et al*., 2019; Wang *et al*., 2019; Gubler *et al*., 2020), including for the European agricultural (Ceglar and Toreti, 2021) and energy sectors (Clark *et al*., 2017). In another example, Scaife *et al*. (2014) showed that GloSea5 provides skilful forecasts for winter NAO up to 4 months ahead. Others have evaluated the dynamical predictions of these two systems. For example, Baker *et al*. (2018a) found that although SEAS5 and GloSea5 both have significant skill in predicting MSLP for the North Atlantic region in wintertime, the skill of GloSea5 is higher in this region. Moreover, previous studies have reported limited skill by dynamical models in directly predicting precipitation (e.g., Scaife *et al*., 2014; Baker *et al*., 2018a; Lledó *et al*., 2020), highlighting the need to post‐process output to reduce systematic model errors (Manzanas *et al*., 2019). Others have combined statistical and dynamical methods for subseasonal to seasonal precipitation and streamflow forecasting (Schepen *et al*., 2012; Strazzo *et al*., 2019). For instance, Baker *et al*. (2018b) used MSLP hindcasts from GloSea5 to compute linear combinations of two MSLP‐based indices for regional forecasting of precipitation in nine UK regions. They found that precipitation forecast skill was improved by using MSLP hindcasts from GloSea5 compared to direct GloSea5 precipitation output.

Despite advances in statistical and dynamical approaches to seasonal forecasting, there have been few assessments of their potential application to Ireland. Situated on the Atlantic margins of Europe with a highly dynamic climate, Ireland offers a stern test of seasonal forecasting capabilities. Recent attempts at seasonal hydrological forecasting highlight the potential value‐added by skilful precipitation forecasts for the water sector, particularly in winter. For example, Foran Quinn *et al*. (2021) assessed the seasonal forecast skill of persistence‐based methods applied to river flows in 46 Irish catchments. They found that skill was greatest when initialized in summer months in catchments with significant groundwater storage. Likewise, Donegan *et al*. (2021) applied an ensemble streamflow prediction (ESP) method and found greatest skill in summer. Also, they showed that by conditioning the ESP with GloSea5 NAO hindcasts, discrimination skill for low flows in winter improved over lead‐times of 1–3 months, particularly during dry winters.

This paper advances the above techniques by evaluating the extent to which hybrid statistical–dynamical methods provide skilful forecasts of winter and summer precipitation across Ireland, over various lead times. Although anomalies in North Atlantic SLP are known to influence climate variability in the UK and Ireland (Comas‐Bru and McDermott, 2014; Hameed and Riaz, 2020), we assess the extent to which hindcasts of North Atlantic MSLP from SEAS5 and GloSea5 can be used as potential predictors in two data‐driven models (linear regression, and an Artificial Neural Network [ANN]). The rest of the paper is organized as follows. Section 2 describes the data used and the framework for deriving climate indices based on MSLP. Section 3 presents the model skill for four rainfall regions and the Island of Ireland, followed by a discussion of the findings and conclusions in section 4.

## 2 DATA AND METHODS

### 2.1 Data

We use output from SEAS5, the latest version of the ECMWF seasonal forecasting system (Johnson *et al*., 2019); and GloSea5, a high‐resolution seasonal forecasting system developed by the UK Met Office (Maidens *et al*., 2013). For SEAS5, we use ensemble hindcasts of MSLP covering the period 1994–2016, consisting of 25 members with 1‐ to 6‐month lead‐times. Similarly, MSLP hindcasts from 28 ensemble members were employed from GloSea5 for the period 1993–2016, also for lead times of 1–6 months. Similar to others, we analyse the ensemble mean hindcasts of SEAS5 and GloSea5 which are known to outperform the median as well as individual ensembles (e.g., Al Samouly *et al*., 2018; Baker *et al*., 2018b; Gubler *et al*., 2020). All SEAS5 and GloSea5 data were obtained from the Climate Data Store of the Copernicus Climate Change Service (https://cds.climate.copernicus.eu/cdsapp#!/dataset/seasonal-monthly-single-levels?tab=overview) at 1° grid resolution (ECMWF, 2019). We apply the same domain as Hall and Hanna (2018), namely 90°W–40°E and 20°–80°N. This area was used to develop the input indices for seasonal forecasting precipitation in winter (DJF) and summer (JJA). In addition to MSLP, we also extracted precipitation hindcasts from GloSea5 and SEAS5 for the Island of Ireland during the period 1993–2016. These data were used to evaluate the value‐added by the statistical step in our modelling framework.

Observed MSLP from ERA5 reanalysis (Hersbach *et al*., 2020) with 0.25° grid resolution was also used. This dataset has served as a reference dataset in previous studies (e.g., Lloyd *et al*., 2018). Observed daily 0.1° grid‐resolution precipitation for the period 1950–2019 was obtained from E‐OBS precipitation (Cornes *et al*., 2018), provided by the European Climate Assessment and Dataset consortium. E‐OBS data were used to derive precipitation regions for the Island of Ireland (section 2.2). This dataset has been used by many researchers to assess other precipitation simulations. For example, Navarro *et al*. (2019) evaluated the performance of IMERG precipitation over Europe with E‐OBS as the reference, while Crhová and Holtanová (2018) compared the outputs from two regional climate models and four global climate models with E‐OBS.

### 2.2 Deriving precipitation regions

**FIGURE 1:**
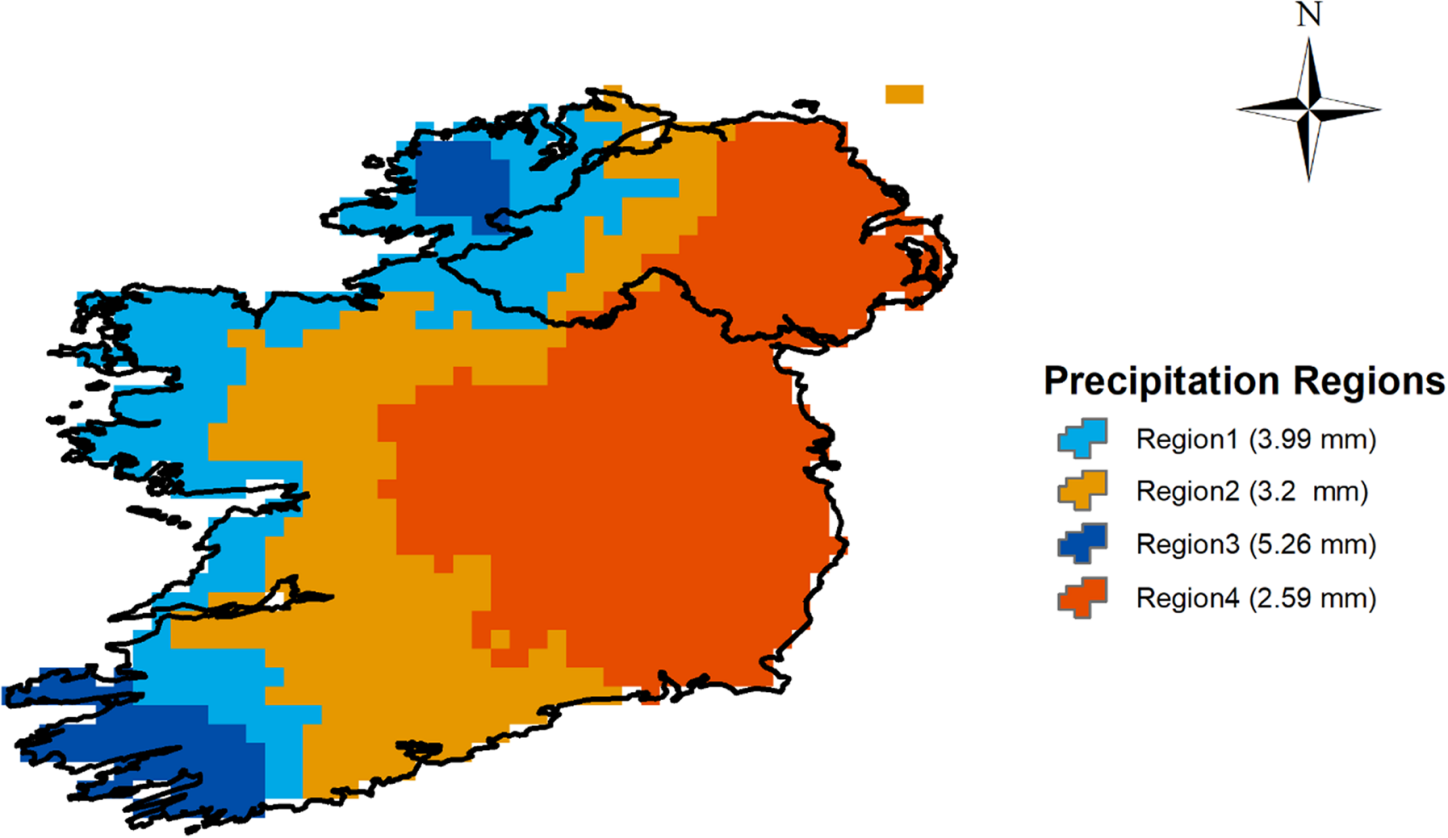
Homogenous regions identified for the Island of Ireland by *K*‐means clustering of EOBS daily precipitation [Colour figure can be viewed at wileyonlinelibrary.com]

To aggregate results and reduce spatial heterogeneity, we derived homogenous precipitation regions to explore the relationship between precipitation and climate signals derived from MSLP. Precipitation regions were derived by *K*‐means clustering of daily E‐OBS data. The number of clusters was determined based on a plot of total within‐group sum of squares versus the number of clusters (not shown). Figure 1 presents the four resulting precipitation regions with daily mean values in parenthesis. Regions 2 and 4 have relatively low precipitation and are in the Midland and East regions of Ireland. Regions 1 and 3 are in the western part of the Island with higher precipitation and greater variation in topography. For each precipitation region, monthly area‐average precipitation series were produced for the period 1993–2016 and used as the target (predictand) in our data‐driven forecast models.

### 2.3 Derivation of MSLP indices

**FIGURE 2:**
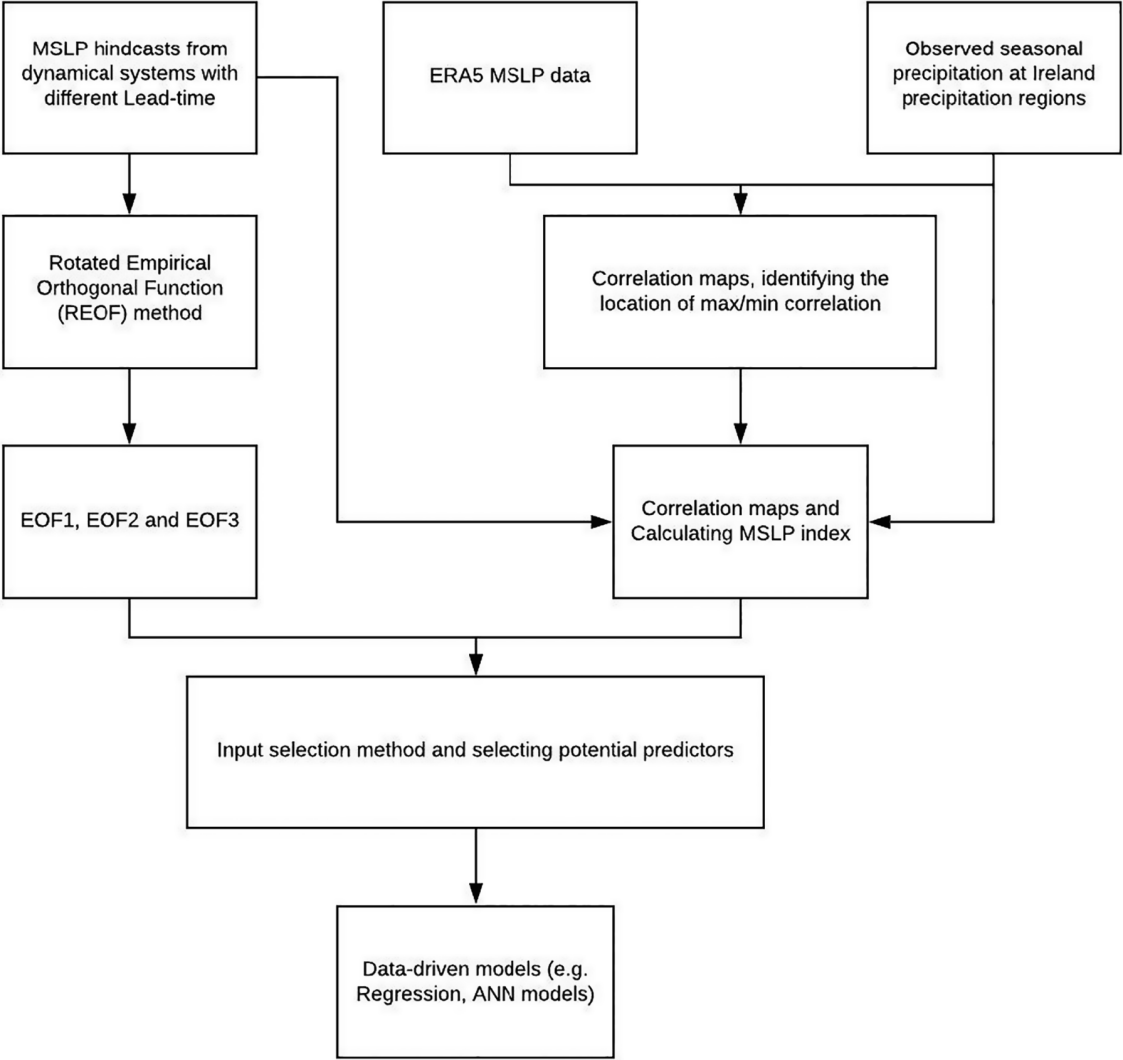
Workflow for the dynamical–statistical approach adopted in this study

Two sets of indices were derived from SEAS5 and GloSea5 MSLP hindcasts and used as predictors for precipitation forecasts. The first follows the simple standardized MSLP index of Baker *et al*. (2018b). The second comprises the three leading components of a rotated empirical orthogonal function (REOF) (Hall and Hanna, 2018; Liu *et al*., 2019) applied to the ensemble mean MSLP hindcasts from each model and lead time. Figure 2 shows the flowchart of the methodology employed. Further details on the indices are provided below.

#### 2.3.1 Standardized MSLP index

To avoid overfitting real relationships by prescreening predictors directly from model output (DelSole and Shukla, 2009), correlation maps for seasonal mean MSLP from ERA5 versus observed precipitation were first generated for each precipitation region to identify locations of maximum and minimum correlation. We used MSLP with the same spatial domain as Baker *et al*. (2018b), that is, 50°W–50°E and 20°–80°N. Like Baker *et al*. (2018b) fixed points of maximum and minimum correlation between MSLP and precipitation from observations are used to derive the standardized MSLP index from model hindcasts for each precipitation region. Depending on the source of the MSLP hindcasts (i.e., GloSea5 or SEAS5), the index ${\text{MSLP}}_{\text{GloSea}5/\text{SEAS}5}$ is defined as the standardized (i.e., centred about the mean and divided by the standard deviation over the time series) MSLP difference between the fixed maximum and minimum points of correlation from ERA5 MSLP (see Equations (1) and (2)). This index was derived for each precipitation region and lead time separately using the ensemble mean,

(1) $${\text{Stand}.\_\text{MSLP}}_{t}^{\max/\min}=\frac{{\text{MSLP}}_{t}-\mu_{\text{MSLP}}}{\sigma_{\text{MSLP}}},$$

(2) $${\text{MSLP}}_{\text{GloSea}5/\text{SEAS}5}={\text{Stand}.\_\text{MSLP}}_{t}^{\max}-{\text{Stand}._{\text{MSLP}}}_{t}^{\min},$$

where t is time (year), ${\text{Stand}.\_\text{MSLP}}_{t}^{\max/\min}$ is the standardized MSLP from GloSea5 or SEAS5 at the maximum or minimum correlation point (derived from ERA5 MSLP) and $\mu_{\text{MSLP}}$ and $\sigma_{\text{MSLP}}$ are the mean and standard deviation of MSLP.

#### 2.3.2 Rotated empirical orthogonal equation indices

To derive the EOF indices we applied a REOF as in Hall and Hanna (2018). Rotated EOF analysis can avoid artificial dipole‐type patterns which can be produced by traditional EOF analysis (Lian and Chen, 2012; Liu *et al*., 2019). Application of REOF to MSLP anomalies was undertaken separately for winter and summer with respect to the long‐term seasonal mean (1993–2016 for GloSea5; 1994–2016 for SEAS5). To account for latitudinal variation in grid cell areas, MSLP anomalies were weighted by the cosine of the latitude prior to analysis. The three leading vectors of the cross‐correlation matrix calculated from monthly MSLP from GloSea5 and SEAS5 hindcasts were used to construct two sets of indices namely $\mathrm{EOF}1_{\text{GloSea}5}$, $\mathrm{EOF}2_{\text{GloSea}5}$, $\mathrm{EOF}3_{\text{GloSea}5}$ and $\mathrm{EOF}1_{\text{SEAS}5}$, $\mathrm{EOF}2_{\text{SEAS}5}$ and $\mathrm{EOF}3_{\text{SEAS}5}$.

### 2.4 Empirical models

Using the MSLP indices described above, an exhaustive search was undertaken of every possible combination of predictors when developing our MLR and ANN models. To avoid overfitting, each combination of predictors was tested for collinearity and predictors selected based on adjusted *R*‐squared (Adj*‐R* ^*2*^) which shows the incremental gain in explained variance for every new predictor included. Adj*‐R* ^2^ is calculated based on the value of *r*‐squared, number of independent variables (predictors), and sample size. Predictors were checked for multicollinearity using the variance inflation factor (VIF) and only those with VIF < 4 were retained for further analysis (cf. Lin, 2008).

Models were developed to predict seasonal precipitation up to 4 months in advance, that is, lead‐times (LT) from one to 4 months (henceforth LT1, LT2, LT3, and LT4). We used MSLP hindcasts from both SEAS5 and GloSea5 for lead times of up to 6 months. Therefore, using the average seasonal MSLP derived from monthly MSLP values, the maximum lead‐time which could be provided is 4 months in advance for the first month of each season, followed by 5‐ and 6‐months lead‐time for the second and third months of that season, respectively. As an example, for winter at four‐month lead time (LT4), MSLP is taken as the average of hindcasts from August (i.e., LT4 for December, LT5 for January and LT6 for February). Table S1, Supporting Information shows the various start months used to calculate seasonal precipitation and MSLP with different lead‐times in winter and summer. Model performance was evaluated using the correlation coefficient (*r*) and mean absolute error (MAE).

#### 2.4.1 Regression models

Multiple linear regression was employed to predict precipitation in different seasons, that is, winter and summer as the target (predictand) with selected MSLP indices from hindcasts as predictors. Regression models were built for each season, lead time, and region (i.e., 2 seasons × 4 lead‐times × 5 regions, yielding 40 models). Using selected predictors, regression models were fitted using leave‐one‐out cross‐validation (LOOCV). In this method, first 1 year is left out and the model is calibrated based on data in other years and the error associated with prediction (of the year omitted from calibration) is recorded. This procedure is repeated for all years and the overall prediction error is computed as the average of all test error estimates.

#### 2.4.2 Artificial neural network

A multilayer perceptron neural network with two hidden layers, trained by backpropagation, was used as the second model for precipitation prediction. The same predictors identified for the MLR method for each region, season and lead time were used. Logistic and linear activation functions were employed for training hidden and output layers, respectively. The optimum number of neurons in each hidden layer was identified via trial‐and‐error. Again, a separate ANN model was built for each season, lead time, and region. Figure S1 shows an example ANN architecture for the case of winter precipitation at LT3 using ${\text{MSLP}}_{\text{GloSea}5}$, $\mathrm{EOF}2_{\text{SEAS}5}$, and $\mathrm{EOF}2_{\text{GloSea}5}$ as the best inputs. Two hidden layers were employed, comprising two neurons for the first hidden‐layer and three for the second. The performance of the ANN model was also assessed using the LOOCV method.

#### 2.4.3 Benchmark models

To evaluate the added skill of the new methods, we apply a zero‐order forecast (ZOF) based on the persistence of observed precipitation (following Dixon and Wilby, 2016). This method assumes that the precipitation at time $\left(t+1\right)$ is the same as at time t (i.e., the previous time step). In addition, the ensemble mean precipitation hindcasts from GloSea5 and SEAS5 for each lead time were first averaged over each precipitation region in Figure 1, then bias‐corrected via empirical quantile mapping (Cannon *et al*., 2015). The results were then compared with precipitation derived from the developed models over those regions. Finally, we derived a climatology benchmark from EOBS for each region using a moving average method. In this case, for each prediction year, the mean precipitation based on a subset of data with length L (window size) up to that year (t) is considered as the predicted precipitation value for that year. Then this averaging window is shifted forward by 1 year to calculate the predicted value at year t+1. We evaluated different window sizes ranging from 5 to 30 years to determine the optimum (i.e., toughest to beat) climatology benchmark based on MAE.

### 2.5 Uncertainty analysis

Having identified the model with most satisfactory performance for each rainfall region, season, and lead time, an uncertainty analysis was conducted using different combinations of input–output data for training–testing the regression and ANN models. This provides an estimate of uncertainty associated with the sampling period, that is, due to climate variability. It should be noted that this does not capture uncertainty among dynamical model ensembles members (because we only consider the ensemble mean as in Baker *et al*., 2018b), nor uncertainties associated with the prediction models themselves, that is, from fitted coefficients of the regression model or the weights of the ANN (see section 4). One thousand random combinations of input–output data were selected to train/test the statistical models for each region‐lead‐time‐season and the resultant forecasted precipitation time series was used to derive 95% confidence intervals of predictions (2.5 and 97.5% percentiles), and to calculate the precipitation with 50% likelihood. Next, *p‐* and *r‐*factors were used to evaluate model performance. The *p‐*factor shows the percentage of observed data bracketed by the 95% uncertainty range. The closer to 1, the better the model performance. The *r*‐factor evaluates the width of the uncertainty band and is calculated using the following equations:

(3) $$r-\text{factor}=\frac{\bar{d_{x}}}{\sigma_{x}},$$

where $\sigma_{x}$ is the standard deviation of variable X (observed precipitation) and $d_{x}$ is calculated using following equation:

(4) $$d_{x}=\frac{1}{n}\sum_{i=1}^{n}\left(X_{U}-X_{L}\right),$$

where n is number of observed data, $X_{U}$ and $X_{L}$ are upper (97.5%) and lower (2.5%) boundaries of uncertainty band. Again, an r−factor value closer to 1 is desirable (Abbaspour, 2008).

## 3 RESULTS

### 3.1 Correlation analysis

**FIGURE 3:**
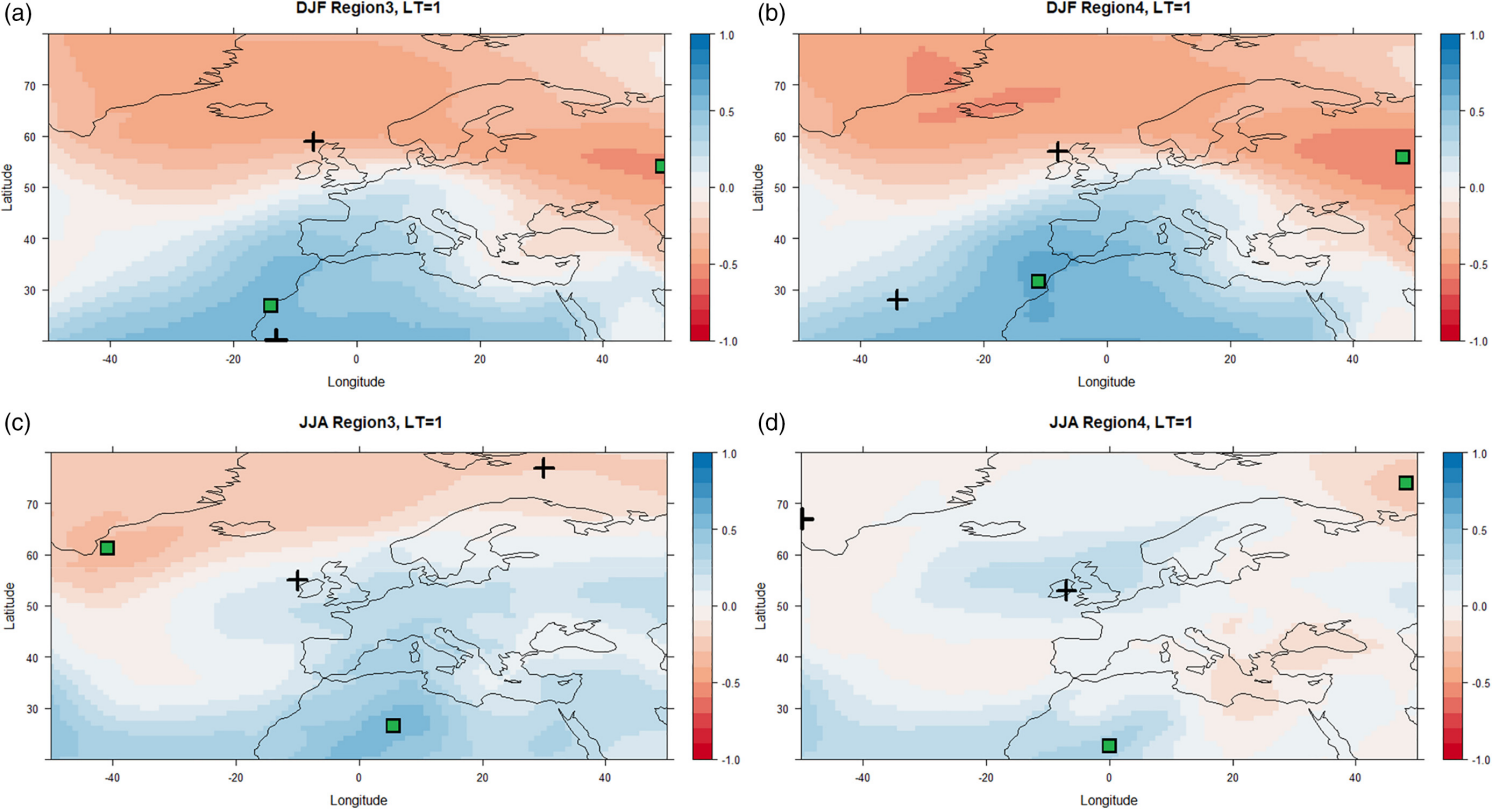
Correlation surfaces for winter (DJF) MSLP with 1‐month lead‐time and winter precipitation (a, b) and summer (JJA) MSLP with 1‐month lead‐time and summer precipitation (c, d) based on GloSea5 for the period 1994–2016 for Regions 3 and 4. Crosses show the location of maximum and minimum correlation values in each case calculated using the ERA5 MSLP dataset. Green squares show the location of max/min correlation between models and observations (GloSea5 MSLP v E‐OBS precipitation) [Colour figure can be viewed at wileyonlinelibrary.com]

**FIGURE 4:**
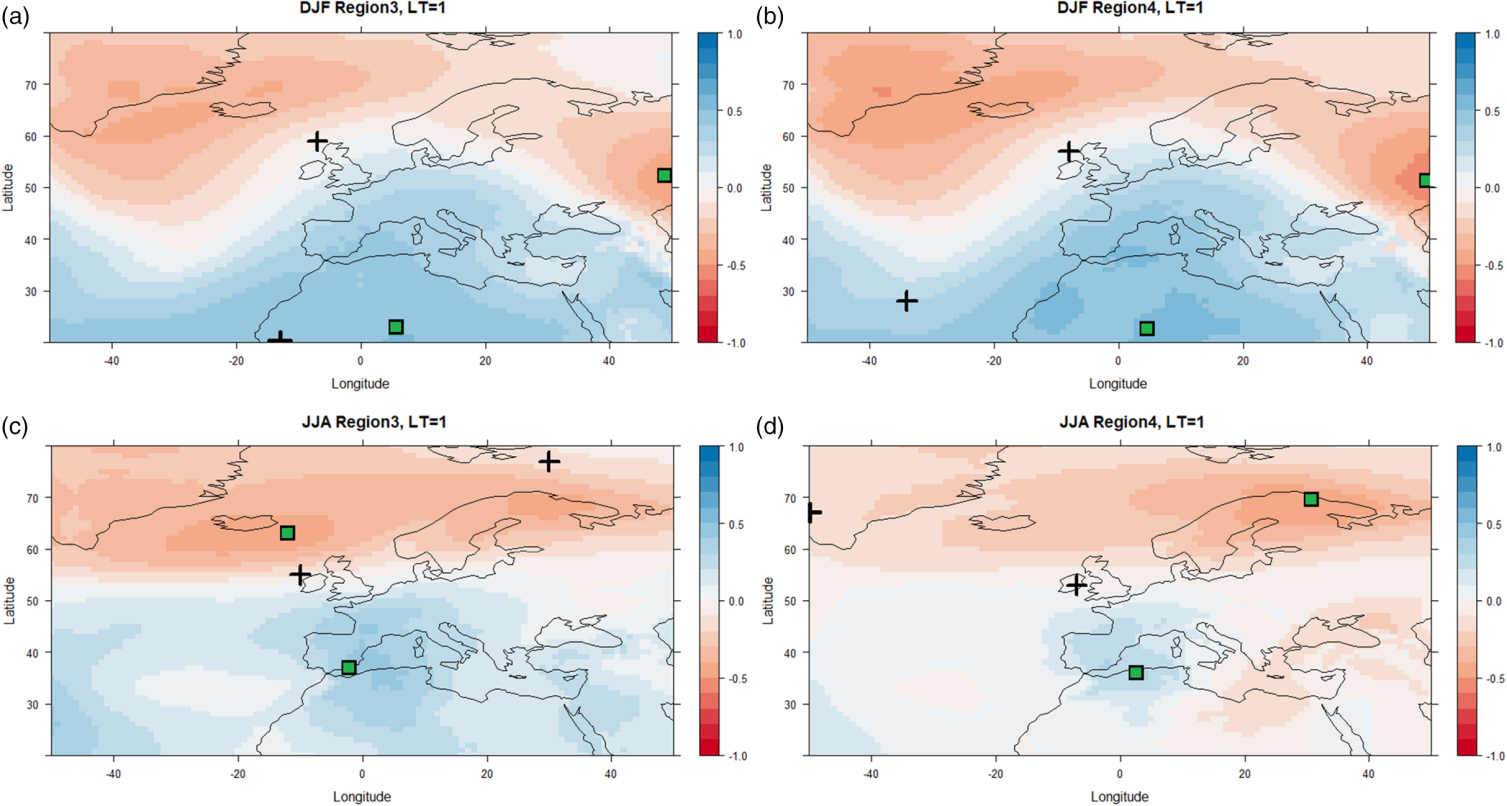
As in Figure 3 but for MSLP from SEAS5 [Colour figure can be viewed at wileyonlinelibrary.com]

The correlation between MSLP and precipitation was found to be similar between regions, but varies by lead time and season and also depends on the dynamical model. Figure 3 shows example correlation surfaces for observed winter and summer precipitation versus MSLP with 1‐month lead‐time from GloSea5 for Regions 3 and 4 as the wettest and driest regions, respectively. Figure 4 shows the same information but for SEAS5. Plus signs denote the locations of maximum and minimum correlation values for each case based on observed MSLP from ERA5. The correlation patterns for GloSea5 and SEAS5 are most similar in winter for LT = 1 (Figures 3a,b and 4a,b) but differ in summer (Figures 3c,d and 4c,d). The MSLP values at maximum and minimum correlation points on each map were used to calculate the standardized MSLP‐based indices for each lead‐time and region. Table S2 shows the correlation ranges obtained for GloSea5 and SEAS5 derived from standardized MSLP indices and precipitation across all regions, lead times, and seasons. The leading EOF patterns derived from GloSea5 and SEAS5 for the different seasons and 1‐month lead‐time are shown in Figures S4–S7, respectively.

**FIGURE 5:**
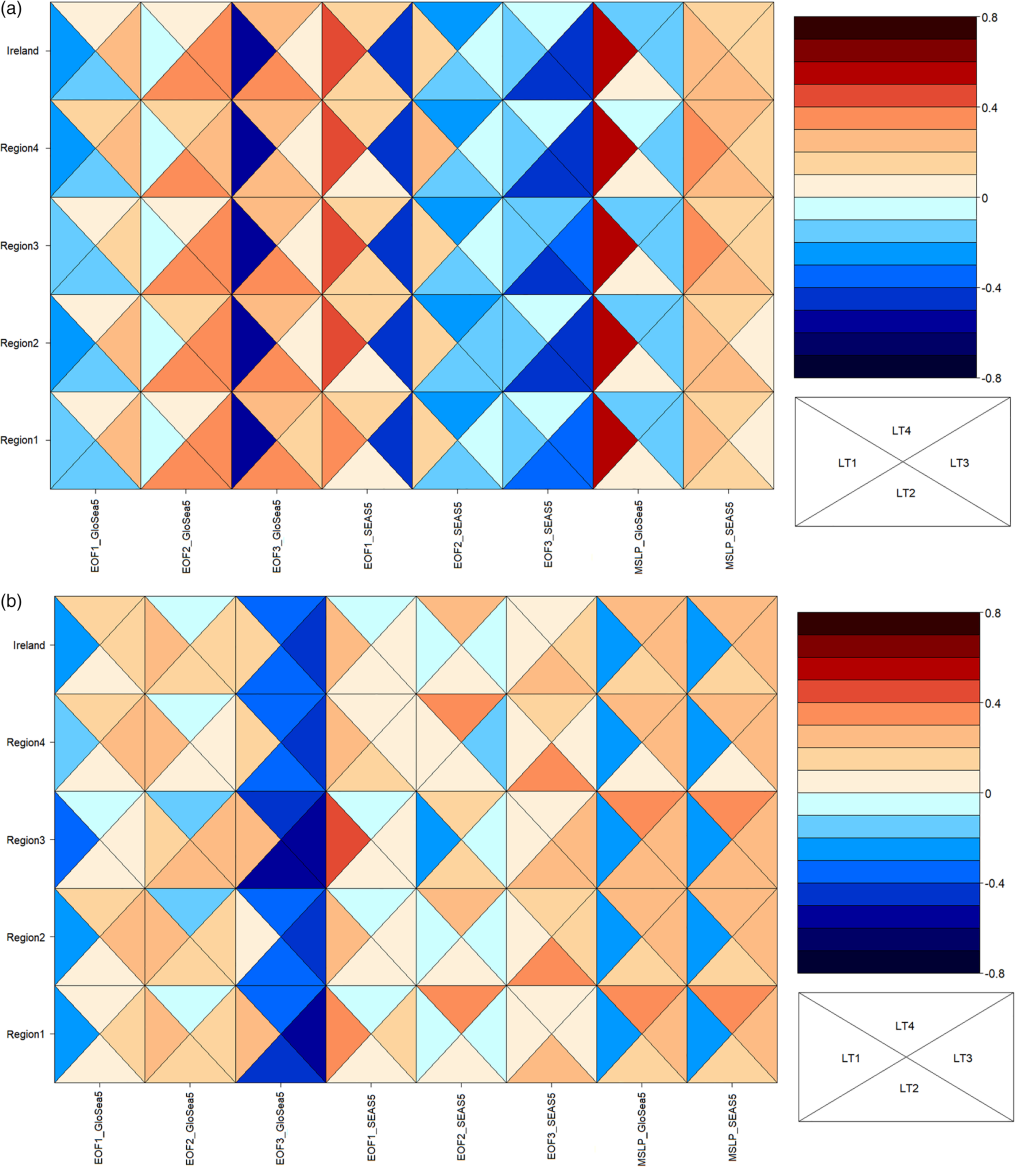
Correlations between dynamical model MSLP EOF indices and precipitation by lead time (LT) and region in (a) winter and (b) summer [Colour figure can be viewed at wileyonlinelibrary.com]

Correlation analysis between standardized MSLP indices/EOFs and observed precipitation over each season, lead‐time, region, and MSLP product was performed to assess how well those potential predictors are related to observed precipitation. The results are shown in Figure 5. For all regions in winter, ${\text{MSLP}}_{\text{GloSea}5}$ and EOF3 from GloSea5 have significant (*p* = .05) positive and negative correlations with winter precipitation, respectively and are considered as potential predictors for regression models for 1‐month lead‐time. For the 2‐month lead‐time in winter, the strongest correlations between observed precipitation in each region are for EOF3 from SEAS5 (*r* < −0.39 for all regions) and for EOF2 from GloSea5 (*r* > 0.33), respectively. For 3‐month lead‐time in winter, EOF1 (*r* < −0.42) and EOF3 (*r* < −0.39) from SEAS5 show significant negative correlations and are, therefore, candidates for precipitation prediction over all regions, together with EOF2 from GloSea5 (*r* > 0.30). For LT4, the strongest positive and negative significant correlations are evident for EOF3 from GloSea5 and EOF2 from SEAS5, respectively.

Summer correlations show greater variation by lead time and between regions (Figure 5b). For some lead‐times, multiple highly correlated indices which could be a promising predictor for most regions are evident but at other lead‐times few predictors with significant correlation with precipitation are found. For 1‐month lead‐time, EOF1 from GloSea5 (*r* < −0.18), ${\text{MSLP}}_{\text{GloSea}5}$ (*r* < −0.23) and ${\text{MSLP}}_{\text{SEAS}5}$ (*r* < −0.23) show significant negative correlations and are potential predictors for statistical models in all regions. For the 2‐month lead‐time, EOF3 from GloSea5 has the strongest negative correlation with summer precipitation over all regions (*r* > −0.33), while EOF3 from SEAS5 showed the strongest significant positive correlations (*r* > 0.22) among all signals. For the 3‐month lead‐time, again EOF3 from GloSea5 (*r* < −0.43) and ${\text{MSLP}}_{\text{GloSea}5}$ and ${\text{MSLP}}_{\text{SEAS}5}$ can be used as a potential predictor for all regions. Finally, for the 4‐month lead‐time, EOF3 from GloSea5 for all regions (*r* < −0.34) and ${\text{MSLP}}_{\text{GloSea}5}$ and ${\text{MSLP}}_{\text{SEAS}5}$ may be useful candidates for statistical models to predict summer precipitation, returning statistically significant correlations at 5% significance level.

Previous research (e.g., Moore *et al*., 2013; Tošić *et al*., 2014), found that EOF1 is associated with the NAO in both winter and summer, whereas EOF2 represents the East Atlantic (EA) pattern which is more prominent in winter, comprising a north–south dipole of anomalies. The EA pattern resembles the NAO, but with pressure anomaly centres displaced southeastward and thus is sometimes interpreted as a “southward shifted” NAO (Barnston and Livezey, 1987). Some EOFs derived from the MSLP hindcasts of GloSea5 and SEAS5 do not show physical resemblance to associated climate signals, that is, NAO or EA. It has previously been observed that when different MSLP datasets are used, and due to the constraining (orthogonal) nature of the EOF analysis, patterns may vary markedly between models (Walz *et al*., 2018). Moreover, in similar studies in which EOFs are connected to large climate signals, long‐term sea level pressure data have been used (e.g., MSLP data from 1925 to 1977 (Trenberth and Paolino Jr, 1981); 1900–2010 (Parker *et al*., 2019)). We used MSLP hindcast from 1994 to 2016 which might lead to different patterns or displacement of centres of action. Also using the ensemble mean can lead to differences as the observed EOFs contain unpredictable as well as predictable components. This might be another reason for absence of physically interpretable EOFs in some cases.

### 3.2 Forecast performance

To assess the added value of using EOFs in our analyses, two sets of potential predictors are considered when developing empirical models. First, only MSLP‐based indices are used as input to the models. Second, we used all potential predictors including MSLP‐based and EOFs from GloSea5 and SEAS5 (Figures S8 and S9). An exhaustive search method was employed to identify the best combination of predictors for each region and lead‐time based on all predictors with significant correlation with precipitation. Evidently, utilizing EOFs alongside MSLP‐based indices does improve forecast skill in most regions and lead‐times (Figure S8). Consequently, we retain all potential predictors. Table 1 summarizes the selected predictors for each model (i.e., by region, season, and lead‐time). EOFs from SEAS5 were selected as predictors in data‐driven models for most regions, lead‐times, and seasons. The optimum architecture identified for each ANN is presented in Table S3. A sensitivity analysis of the length of moving average window for the climatology benchmark (Figure S10) determined that a 30‐year window minimizes MAE in both summer and winter across all regions and was thus employed.

**TABLE 1:** The predictors selected for regression and ANN models by lead time (LT), season, and region

		Lead‐time			
		LT = 1	LT = 2	LT = 3	LT = 4
Winter	Region1	${\text{MSLP}}_{\text{GloSea}5}$ + EOF3_SEAS5+ EOF3_GloSea5	EOF1_SEAS5 + EOF2_SEAS5	$\mathrm{EOF}1_{\text{SEAS}5}+\mathrm{EOF}2_{\text{SEAS}5}$ + EOF3_SEAS5	EOF2_GloSea5 + EOF3_SEAS5
	Region2	${\text{MSLP}}_{\text{GloSea}5}$ + ${\text{MSLP}}_{\text{SEAS}5}$ + EOF1_SEAS5 + EOF2_SEAS5	${\text{MSLP}}_{\text{GloSea}5}$ + EOF1_SEAS5 + EOF2_SEAS5	${\text{MSLP}}_{\text{GloSea}5}$ + EOF3_GloSea5 + EOF1_SEAS5+EOF2_SEAS5 + EOF3_SEAS5	EOF2_GloSea5 + EOF3_SEAS5
	Region3	${\text{MSLP}}_{\text{GloSea}5}$ + ${\text{MSLP}}_{\text{SEAS}5}$ + EOF1_SEAS5 + EOF2_SEAS5	$\mathrm{EOF}2_{\text{GloSea}5}+\mathrm{EOF}1\_\text{SEAS}5$ + EOF2_SEAS5	EOF1_SEAS5 + EOF2_SEAS5 + EOF3_SEAS5	EOF2_GloSea5 + EOF2_SEAS5
	Region4	${\text{MSLP}}_{\text{GloSea}5}$ + ${\text{MSLP}}_{\text{SEAS}5}$ + EOF1_SEAS5 + EOF2_SEAS5	$\mathrm{EOF}1_{\text{SEAS}5}+\mathrm{EOF}2\_\text{SEAS}5\;$	${\text{MSLP}}_{\text{GloSea}5}$ + EOF3_GloSea5 + EOF1_SEAS5+EOF2_SEAS5 + EOF3_SEAS5	EOF2_GloSea5 + EOF2_SEAS5
	Ireland	${\text{MSLP}}_{\text{GloSea}5}$ + EOF3_GloSea5 + EOF3_SEAS5	$\mathrm{EOF}1_{\text{SEAS}5}+\mathrm{EOF}2\_\text{SEAS}5\;$	EOF1_SEAS5 + EOF2_SEAS5 + EOF3_SEAS5	EOF2_GloSea5 + EOF2_SEAS5
Summer	Region1	$\mathrm{EOF}2_{\text{GloSea}5}+\mathrm{EOF}1\_\text{SEAS}5$	${\text{MSLP}}_{\text{SEAS}5}$ + EOF1_GloSea5 + EOF3_GloSea5+EOF3_SEAS5	${\text{MSLP}}_{\text{GloSea}5}$ + EOF1_GloSea5+EOF2_GloSea5 + EOF3_GloSea5 + EOF2_SEAS5	${\text{MSLP}}_{\text{GloSea}5}$ + EOF1_GloSea5 + EOF3_GloSea5++EOF1_SEAS5 + EOF3_SEAS5
	Region2	${\text{MSLP}}_{\text{SEAS}5}$ + EOF1_SEAS5	${\text{MSLP}}_{\text{SEAS}5}$ + EOF1_GloSea5 + EOF3_GloSea5+EOF3_SEAS5	${\text{MSLP}}_{\text{GloSea}5}$ + EOF1_GloSea5 + EOF2_GloSea5+EOF3_GloSea5 + EOF2_SEAS5	${\text{MSLP}}_{\text{GloSea}5}$ + EOF2_GloSea5 + EOF3_GloSea5++EOF1_SEAS5 + EOF2_SEAS5
	Region3	${\text{MSLP}}_{\text{SEAS}5}$ + EOF1_SEAS5 + EOF2_SEAS5	${\text{MSLP}}_{\text{SEAS}5}$ + EOF1_GloSea5 + EOF3_GloSea5+EOF1_SEAS5 + EOF3_SEAS5	${\text{MSLP}}_{\text{SEAS}5}$ + EOF2_GloSea5+EOF3_GloSea5 + EOF2_SEAS5 + EOF3_SEAS5	${\text{MSLP}}_{\text{GloSea}5}$ + EOF2_GloSea5 + EOF2_SEAS5+EOF3_SEAS5
	Region4	${\text{MSLP}}_{\text{GloSea}5}$ + ${\text{MSLP}}_{\text{SEAS}5}$ + EOF2_GloSea5 + EOF1_SEAS5	EOF2_SEAS5+ EOF3_SEAS5	${\text{MSLP}}_{\text{GloSea}5}$ + EOF1_GloSea5 + EOF3_GloSea5+EOF2_SEAS5	${\text{MSLP}}_{\text{GloSea}5}$ + EOF2_GloSea5 + EOF2_SEAS5+EOF2_SEAS5
	Ireland	${\text{MSLP}}_{\text{SEAS}5}$ + EOF1_SEAS5	${\text{MSLP}}_{\text{GloSea}5}$ + ${\text{MSLP}}_{\text{SEAS}5}$ + EOF1_GloSea5+EOF3_GloSea5 + EOF1_SEAS5+EOF3_SEAS5	${\text{MSLP}}_{\text{GloSea}5}$ + EOF1_GloSea5 + EOF2_GloSea5+EOF3_GloSea5 + EOF2_SEAS5	${\text{MSLP}}_{\text{GloSea}5}$ + EOF1_GloSea5 + EOF2_GloSea5+EOF3_GloSea5 + EOF2_SEAS5+EOF3_SEAS5

**FIGURE 6:**
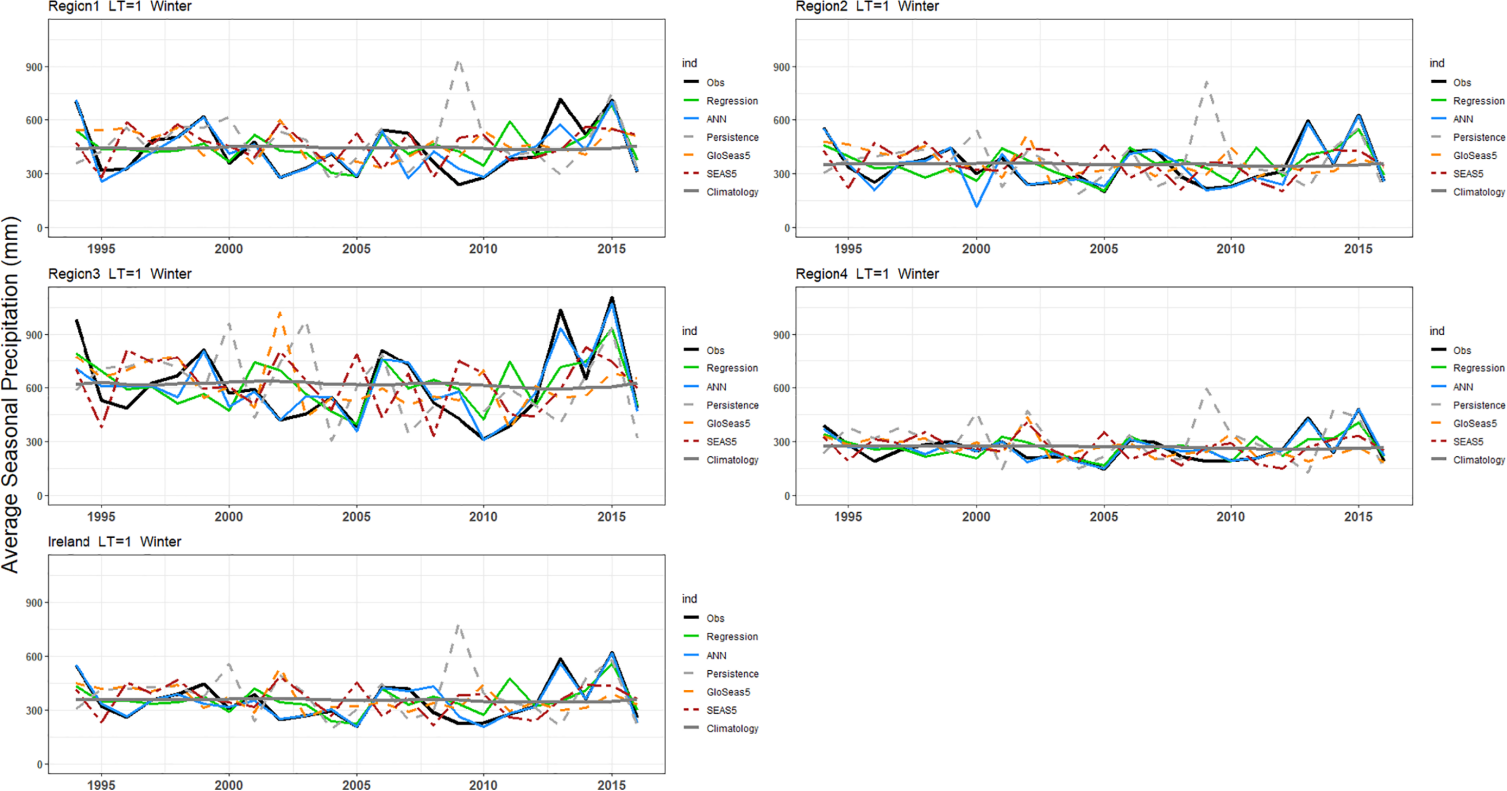
Winter precipitation hindcasted for each region for LT1. Results are shown for EOBS observations (black line), the MLR (green), the ANN (blue), persistence (grey dashed), bias corrected GloSea5 (orange), SEAS5 (red) and climatology (grey) precipitation [Colour figure can be viewed at wileyonlinelibrary.com]

**FIGURE 7:**
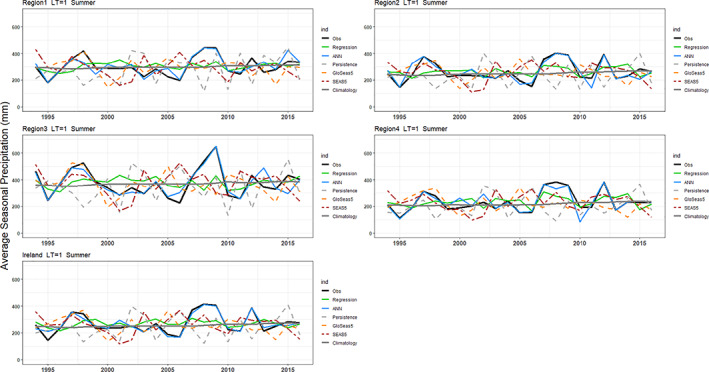
As in Figure 6 but for summer [Colour figure can be viewed at wileyonlinelibrary.com]

Hindcasts for winter and summer precipitation with 1‐month lead‐time are presented in Figures 6 and 7. Overall, for both seasons, regression and ANN hindcasts outperform the persistence, climatology, and bias‐corrected dynamical model output for all regions. Moreover, at the 1‐month lead‐time, the ANN and MLR show some skill at predicting extreme seasons. For example, the dry summer of 1995 and wettest winter on record 2015/2016 are captured well by our hybrid models. Scaife *et al*. (2017) show that the intensified cyclonic flow over the Atlantic in winter 2015/2016 were well predicted by the GloSea5 system. However, the extremeness of the wet 2013/2014 winter and sequence of exceptionally wet summers in 2007–2009 are underestimated (Matthews *et al*., 2014; 2016; Noone *et al*., 2016). Knight *et al*. (2017) assert that the tropics played a significant role in the development of the unusual extratropical circulation that led to widespread high precipitation over the UK in winter 2013–2014.

**FIGURE 8:**
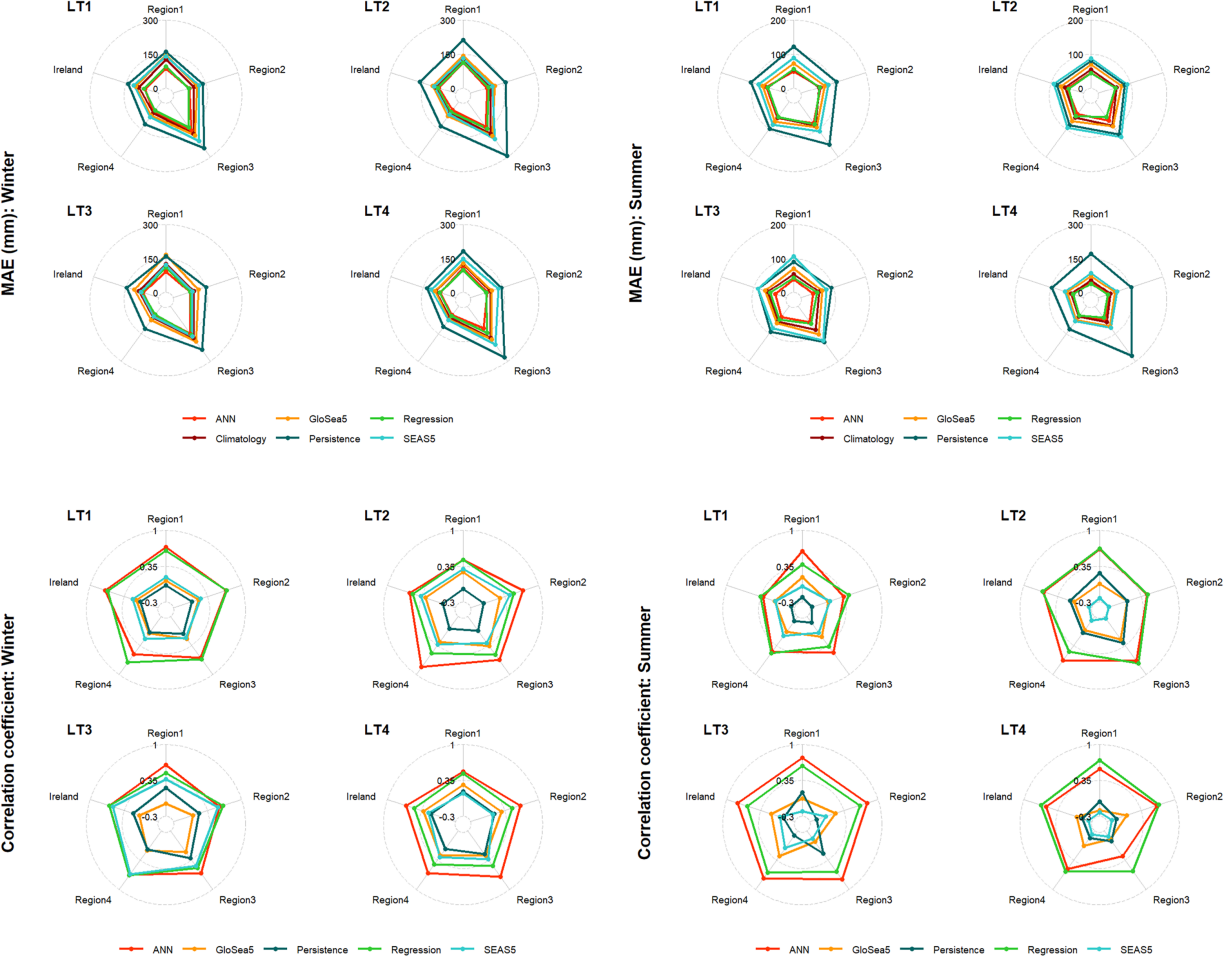
Performance of the methods as evaluated using MAE, and correlation coefficient for different lead‐times (LT) and regions in winter (left column) and summer (right column) [Colour figure can be viewed at wileyonlinelibrary.com]

Performance criteria (i.e., CC and MAE) derived from the different hindcasts across seasons, lead‐times, and regions are shown in Figure 8. For MAE, both the ANN and regression models have the best performance (Figure 8) in winter for all lead‐times compared with other benchmark methods; the ANN outperforms regression at most lead‐times over most regions except LT1 in winter. In this case, the average MAE for all regions is 88 mm for the ANN model, compared with MAE of 85 mm for the MLR model. Except for SEAS5 precipitation in winter for LT3, climatology is the next best performing alterative to the ANN/MLR models. MAE results again show that the persistence method followed by SEAS5 yield the worst performance in winter at lead‐times 1 and 4 and GloSea5 for most regions at lead‐times 2 and 3. In summer, the persistence method followed by SEAS5 has the worst performance in most cases, except at lead‐time 2 where SEAS5 performs worse than the persistence method (Figure 8). It can also be seen that all hindcasts have slightly better performance for Region 4 (driest region) and the weakest performance over Region 3 (wettest region).

Based on the correlation coefficient, again ANN and MLR have most skilful hindcasts for all lead‐times, regions, and seasons with average correlation values of 0.59 and 0.58 for the ANN and 0.50 and 0.56 for MLR in winter and summer, respectively (Figures 8 and S11). The ANN has superior performance in both winter and summer except for LT1 in winter and LT4 in summer; for LT1 and LT2 in summer the ANN and MLR models have very similar performance. In winter, bias‐corrected precipitation from SEAS5 has higher correlation with observations compared to the equivalent from GloSea5 for LT1 and LT3. At LT4, precipitation from GloSea5 performs slightly better over Regions 1 and 2 and Ireland. In summer, the persistence method has higher correlation than GloSea5 and SEAS5 precipitation for LT2.

In summary, our new dynamical–statistical methods perform satisfactorily in prediction of precipitation up to 4 months ahead, surpassing all available benchmarks in both winter and summer. The ANN performs better than MLR in most regions in summer especially for LT1 and LT3, and in winter for LT2, LT3 and LT4. However, the MLR marginally outperforms ANN in most regions at LT1 in winter and LT4 in summer (Figure S11). The skill of bias‐corrected precipitation forecasts from SEAS5 and GloSea5 are generally not as good as climatology in winter and only marginally better than persistence in summer for some lead‐times/regions. For example, the correlations between bias‐corrected SEAS5 and GloSea5 and observed precipitation are −0.001 and 0.06 in winter, and 0.06 and 0.03 in summer, respectively.

To test whether the choice of predictors influences skill over different regions, we generalized the predictors selected for the Island of Ireland to other regions, that is, used identical predictors for all regions. The results are shown in Figure S12 in terms of the correlation coefficient. Using fixed predictors for all regions marginally increases skill in some cases (e.g., ANN in LT1 and ANN and MLR methods in LT4), makes no difference in others (e.g., MLR at LT2 and ANN and MLR methods at LT3) or, in a few cases, decreases the correlation coefficient (e.g., MLR at LT1 over Regions 2, 3, and 4). Based on these results there is no obvious evidence to suggest any systemic overfitting.

### 3.3 Uncertainty analysis

**FIGURE 9:**
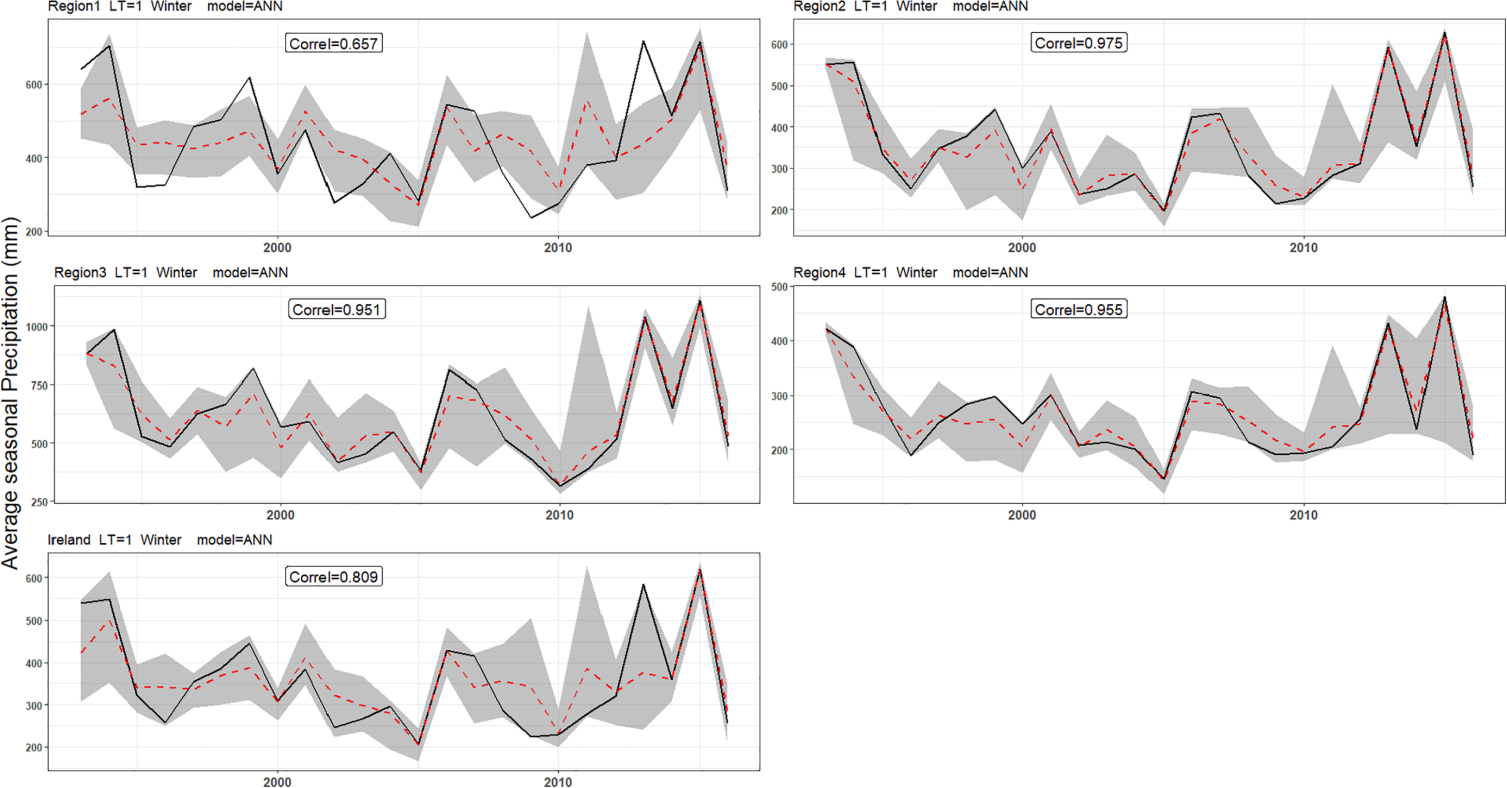
ANN model results for different regions with LT = 1 month in winter, showing the 95% uncertainty band (grey shaded area), the median forecast (red dashed line) and observed precipitation (black line) [Colour figure can be viewed at wileyonlinelibrary.com]

**FIGURE 10:**
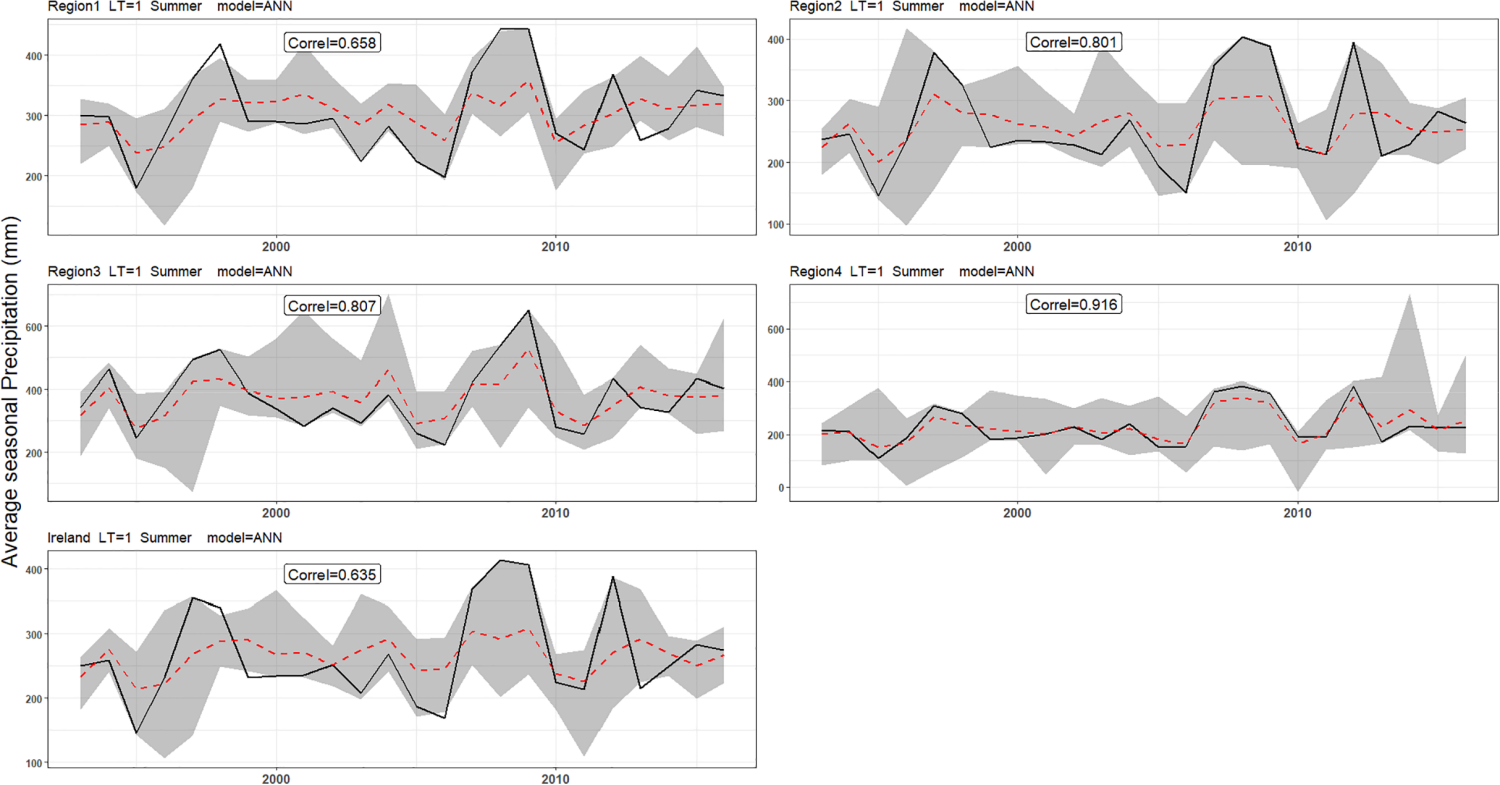
As in Figure 9, but for summer [Colour figure can be viewed at wileyonlinelibrary.com]

Given the consistently strong performance of the ANN method across regions, lead‐times, and seasons, uncertainty due to sampling of calibration period was evaluated by applying different combinations of train‐test periods as input to the ANN. For illustrative purposes, Figures 9 and 10 show 95% confidence intervals (grey area), with the median modelled precipitation (red dashed‐line) and observed precipitation (black line) for different regions and seasons at LT1. It is noteworthy that when varying the calibration period, hindcasts are more successful at capturing extreme seasons. Moreover, the wettest winters (including 2013/14 and 2015/16) are better captured by hindcasts at LT1 and LT3 than other lead‐times. Table 2 shows the p−factor and r−factor values for each region and lead‐time. Sampling uncertainty associated with ANN model forecasts is greater in summer for all lead‐times (i.e., there are larger *r*‐factors in summer compared to winter). However, the uncertainty band brackets more observed precipitation in summer than winter (as shown by the *p*‐factors) for most lead‐times and regions. Although all regions have less uncertainty in winter than summer, more of the observations lie within the uncertainty band of the LT1 forecast (i.e., greater *p*‐factor in winter compared to summer) in Regions 2 and 3 and Ireland. For LT2, the uncertainty in winter is less than summer while a higher proportion of the observations are bracketed by the 95% uncertainty band in summer compared to winter, except in Region 3. Finally, for LT4, sampling uncertainty associated with summer is greater than winter, while more data are bracketed by the uncertainty band in summer in all regions.

**TABLE 2:** Summary of *r*‐ and *p*‐factors associated with the uncertainty in application of the ANN model for each lead time (LT), region and season

	Region	Winter				Summer			
		LT1	LT2	LT3	LT4	LT1	LT2	LT3	LT4
*r*‐factor	Region1	1.05	1.21	1.48	1.13	1.41	1.94	1.96	1.92
	Region2	0.96	0.87	1.04	0.99	1.67	2.51	1.98	1.64
	Region3	1.07	1.43	1.37	0.95	1.97	1.71	1.82	2.14
	Region4	1.07	0.82	1.19	0.76	2.65	1.85	2.05	1.63
	Ireland	1.18	0.91	1.33	0.93	1.49	2.54	2.03	1.92
*p*‐factor	Region1	0.59	0.88	0.99	0.75	0.79	0.92	1	1
	Region2	0.99	0.51	0.99	0.71	0.83	1	1	1
	Region3	0.98	0.99	0.99	0.76	0.96	0.88	0.96	1
	Region4	0.96	0.38	0.96	0.58	1	0.96	1	1
	Ireland	0.92	0.55	0.96	0.77	0.75	1	1	1

## 4 DISCUSSION AND CONCLUSION

We developed hybrid dynamical–statistical approaches to seasonal forecasting of winter and summer precipitation for Ireland over lead times of 1–4 months using hindcasts of MSLP from two dynamical forecasting systems (SEAS5 and GloSea5). We used MLR and ANNs to relate MSLP to observed precipitation over four distinct rainfall regions as well as averaged across the Island of Ireland as a whole. MSLP hindcasts from SEAS5 and GloSEA5 were postprocessed in two ways before being used as potential predictors in our statistical modelling approach. First, we identified the locations of grids with most positive and negative correlations based on ERA5 MSLP (over the period 1993/1994–2016) when correlated with observed (EOBS) precipitation for each region and season. Next, standardized indices of MSLP anomalies were derived from hindcasts of MSLP from dynamical models using the same fixed max/min points of correlation specified for each precipitation region, season, and lead‐time. Second, we deployed REOF analysis to hindcasts of MSLP from SEAS5 and GloSea5 to identify the three leading components for each forecast horizon. We evaluated the performance of this dynamical–statistical approach against bias‐corrected precipitation forecasts from SEAS5 and GloSea5, all benchmarked against skill from persistence and climatology alone.

As with Baker *et al*. (2018b), we find that our dynamical–statistical approach improves forecast skill relative to bias‐corrected, dynamical forecast system output. Our ANN and MLR approaches provide greatest skill for all lead times and regions in both summer and winter; they also outperform precipitation from dynamical models, climatology, and persistence. Consistent with Nobakht *et al*. (2021), precipitation forecasts from SEAS5 performed marginally better than GloSea5, except for LT4 in winter over Regions 1 and 2. Although we used a relatively simple bias correction method, future research might achieve greater accuracy via more sophisticated post‐processing methods, such as quantile delta mapping (Cannon *et al*., 2015; Mendez *et al*., 2020).

Among the potential predictors available, those based on standardized MSLP showed strongest correlations (spanning 0.35–0.63) with precipitation across both seasons, all lead times and regions. This highlights that indices based on MSLP provide a reliable basis for forecasting precipitation over the Island of Ireland, especially in winter. MLSP might also enhance seasonal forecast skill for other climate variables and regions where there is covariance with precipitation, such as for atmospheric humidity, air temperature, sunshine hours and wind speeds (e.g., Hillier *et al*., 2020).

Interestingly, some of the strongest correlations were found between summer precipitation and EOF3 from GloSea5 data (EOF3_GloSea5), with this index selected as a predictor for many regions/lead‐times. This EOF is usually interpreted as the East Atlantic (EA) pattern (Hall and Hanna, 2018), and has also been used in some weather generators as a predictor variable (ATKINS, 2020). Hence, although the NAO has long been recognized as a major driver of climate variability in Northwest Europe, we note that an apparent EA pattern emerges as a key signal of summertime predictability in the dynamical models. EOF3 from SEAS5 also showed strong negative correlation with precipitation in most regions in winter. However, while for some lead‐times the EOFs from GloSea5 and SEAS5 are physically interpretable (e.g., EOF1 from GloSea5 in winter and EOF1 from SEAS5 in summer are both similar to NAO), others are not physically interpretable. For example summer EOF3 from GloSea5 is somewhat similar to Scandinavian pattern (SCA) but the negative centre has a more northeasterly position. Undoubtedly, the physical interpretability is hampered by the brevity of available hindcast MSLP data. Moreover, the MSLP data used for EOF analysis are raw hindcasts from dynamical models, not observations. We also used the ensemble mean rather than ensemble members which could further explain the unfamiliar EOF patterns.

The dynamical–statistical approach developed here leverages two forecasting systems to develop prototype predictions of winter and summer precipitation with up to 4 months lead for regions across the Island of Ireland. Our ANN and regression models show the most consistently better results compared with bias corrected dynamical outputs, climatology, and persistence methods. The possibility of model overfitting was reduced by applying leave‐one‐out cross validation, use of adjusted *R*‐square when evaluating different sets of predictors, as well as use of a smaller set of predictors in all models (Figure S12). Although our dynamical–statistical models performed well in both winter and summer, the ANN provided marginally higher skill in winter, and the MLR in summer. Whereas the ANN and MLR models revealed variability in skill for the wettest rainfall regions in winter and the driest in summer, the MLR model returned most skilful and consistent results in summer for LT1, LT2, and LT4 with average correlation scores of 0.39, 0.59, and 0.64, respectively. However, the ANN model achieved more skilful and consistent results in winter across most regions and lead times, with an average correlation coefficient of 0.61 over all regions for LT1. In comparison, Baker *et al*. (2018b) obtained correlations up to 0.70 between regression‐based hindcasts and observed precipitation in winter for the UK. The encouraging performance of these models, particularly in summer, is noteworthy and paves the way to improved drought forecasting in summer and winter. Previous studies in the region have primarily focused on winter predictions (e.g., Scaife *et al*., 2014; Baker *et al*., 2018b; Stringer *et al*., 2020) or the evaluation of predictors that could be used for summer precipitation/temperature forecasts but without evaluating predictive skill in summer (e.g., Fowler and Kilsby, 2002). Also as Knight *et al*. (2017) reported a significant role of tropical circulation in high precipitation totals over the UK, for example, in winter 2013–2014, in future studies the domain to explore MSLP might be expanded to include tropical regions. Finally, as the availability of hindcasts increases future work may also further assess model performances using out of sample tests for more recent years.

Our findings demonstrate the feasibility of skilful seasonal forecasts of winter and summer precipitation in Ireland. Such forecasts could be of value to many sectors, not least the water industry. However, before evaluating their operational utility, future work should extend the analysis of uncertainty presented here. For example, individual ensemble members rather than just the ensemble mean could be used to generate probabilistic seasonal forecasts. The uncertainty analysis framework used here evaluated uncertainties due to climate variability but not from the dynamical model ensembles. In addition, uncertainties from the statistical models themselves (i.e., the uncertainty in fitted coefficients of regression model and weights of the ANN) were not included. Moreover, predictor–predictand relationships were treated as stationary in this research; for operational purposes the ANN/regression models should be re‐calibrated using a moving window to capture any evolution in the relationships. There is also the possibility of assessing other candidate predictors and domains in future work. For instance, bias‐corrected precipitation forecasts from dynamical systems could be used as inputs to data‐driven models alongside the MSLP‐based indices employed here.

## AUTHOR CONTRIBUTIONS

**Saeed Golian:** Conceptualization; formal analysis; methodology; software; supervision; validation; visualization; writing – original draft; writing – review and editing. **Conor Murphy:** Conceptualization; methodology; project administration; supervision; validation; writing – review and editing. **Robert L. Wilby:** Methodology; validation; writing – review and editing. **Tom Matthews:** Methodology; validation; writing – review and editing. **Seán Donegan:** Data curation; writing – review and editing. **Dáire Foran Quinn:** Data curation; resources. **Shaun Harrigan:** Methodology; validation; writing – review and editing.

## Supporting information


**Appendix** S1: Supporting information
