# Supplementary material for: Dynamical–statistical seasonal forecasts of winter and summer precipitation for the Island of Ireland
Source: Int J Climatol. 2022 Feb 15;42(11):5714–31. doi: 10.1002/joc.7557 (PMC9540122; doi:10.1002/joc.7557)
Supplement: Supplementary file 1 — Appendix S1: Supporting information [file JOC-42-5714-s001.docx]

**Supplementary Information**

**Table S1 Monthly hindcasts used to derive seasonal average MSLP and precipitation for LT1 to LT4 in winter [DJF] and summer [JJA]. For example, for winter LT1, MSLP is taken as the average of hindcasts from November at LT1 (December), LT2 (January) and LT3 (February). Winter LT2 takes hindcasts of each winter month from October and so on to winter four month lead time which takes August hindcasts at LT4 (December), LT5 (January) and LT6 (February).**

|  | Lead-Time | Winter | Summer |
| --- | --- | --- | --- |
|  | LT1 | Nov(LT1)+Nov(LT2)+Nov(LT3) | May(LT1)+May(LT2)+May(LT3) |
|  | LT2 | Oct(LT2)+Oct(LT3)+Oct(LT4) | Apr(LT2)+Apr(LT3)+Apr(LT4) |
|  | LT3 | Sep(LT3)+Sep(LT4)+Sep(LT5) | Mar(LT3)+Mar(LT4)+Mar(LT5) |
|  | LT4 | Aug(LT4)+Aug(LT5)+Aug(LT6) | Feb(LT4)+Feb(LT5)+Feb(LT6) |

**Table S2 Correlation range across all regions between standardized MSLP indices and precipitation for different lead-times and seasons for GloSea5 and SEAS5.**

|  | GloSea5 | | SEAS5 | |
| --- | --- | --- | --- | --- |
| Lead-time | Summer | Winter | Summer | Winter |
| LT1 | -0.27- -0.23 | 0.51-0.56 | -0.27- -0.23 | 0.22-0.33 |
| LT2 | 0.03-0.24 | 0.01-0.08 | 0.03-0.24 | 0.14-0.26 |
| LT3 | 0.22-0.29 | -0.12- -0.10 | 0.22-0.29 | 0.03-0.18 |
| LT4 | 0.22-0.32 | -0.16- -0.08 | 0.22-0.32 | 0.11-0.21 |

**Table S3 The selected ANN architecture for different lead-times, seasons and regions. The first and second number in bracket are the number of neurons in the first and second hidden layers, respectively.**

|  | Winter | | | | Summer | | | |
| --- | --- | --- | --- | --- | --- | --- | --- | --- |
| Region | LT1 | LT2 | LT3 | LT4 | LT1 | LT2 | LT3 | LT4 |
| Region1 | (3,4) | (11,4) | (4,3) | (9,9) | (10,9) | (13,4) | (14,8) | (10,10) |
| Region2 | (15,4) | (14,4) | (9,6) | (11,8) | (15,8) | (10,5) | (13,9) | (13,4) |
| Region3 | (9,8) | (14,6) | (8,7) | (13,6) | (13,3) | (11,5) | (11,6) | (6,5) |
| Region4 | (11,7) | (13,3) | (7,9) | (14,8) | (12,5) | (15,9) | (8,7) | (12,4) |
| Ireland | (12,9) | (13,4) | (5,3) | (14,10) | (11,6) | (12,8) | (15,7) | (11,7) |


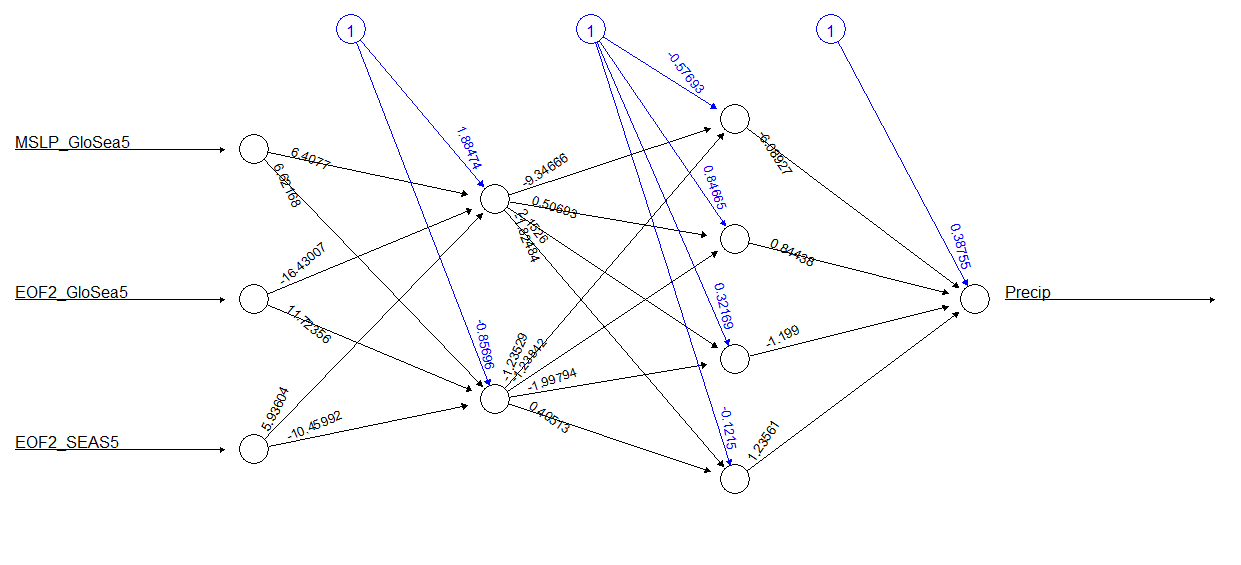


**Figure S1 Example ANN architecture for predicting winter precipitation in Region 3 at LT3**


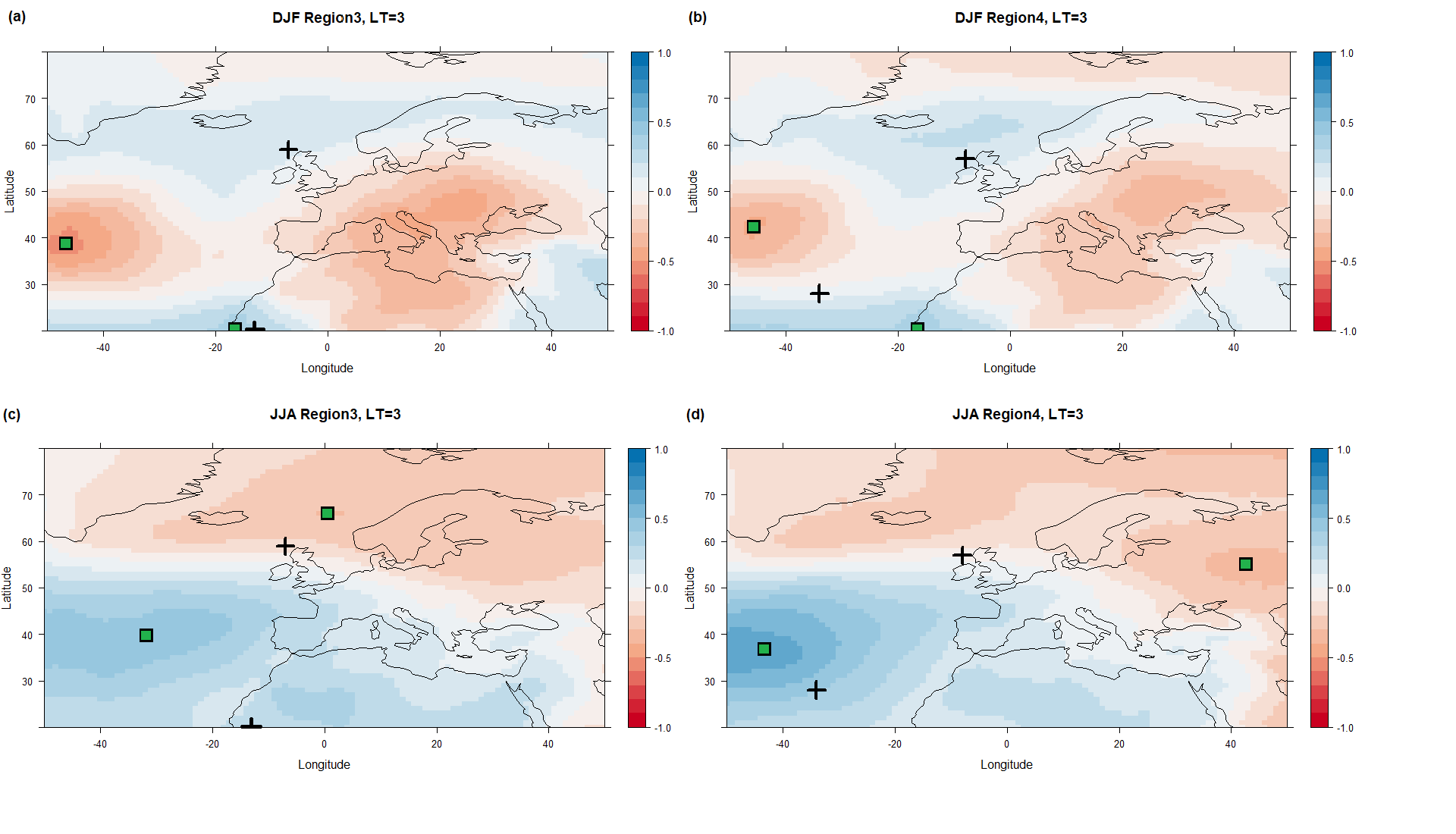


**Figure S2 Correlation surfaces for winter [DJF] MSLP at LT3 and winter precipitation (a and b) and summer [JJA] MSLP at LT3 and summer precipitation (c and d) from GloSea5 for the period 1994–2016 for Regions 3 and 4. Black crosses show the location of max/min corration based on quasi observations (ERA5 MSLP v E-OBS precipitation) and green squares show the location of max/min correlation between models and observations (GloSea5 MSLP v E-OBS precipitation)**


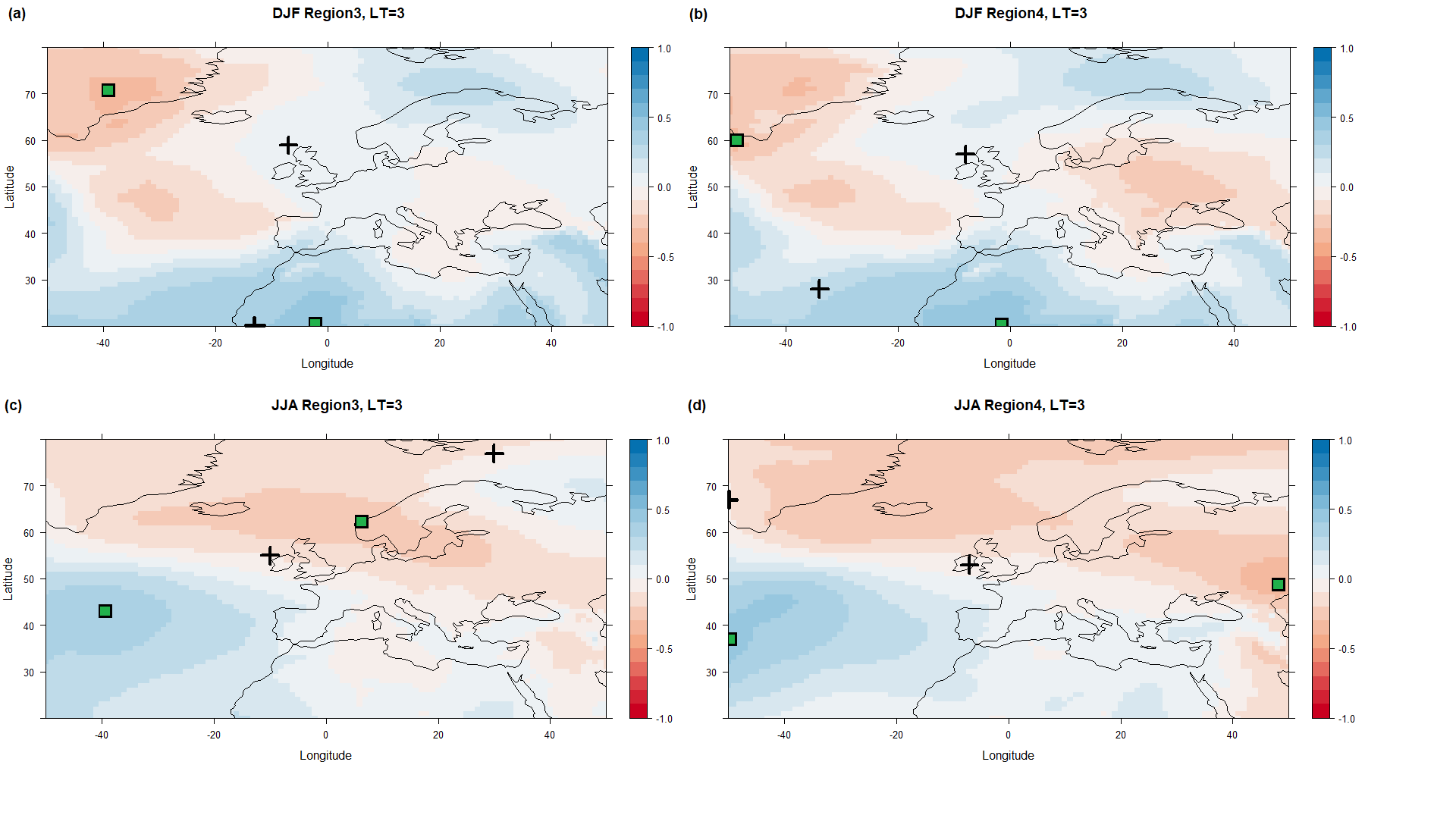


**Figure S3 As in Figure S2 but for MSLP from SEAS5.**


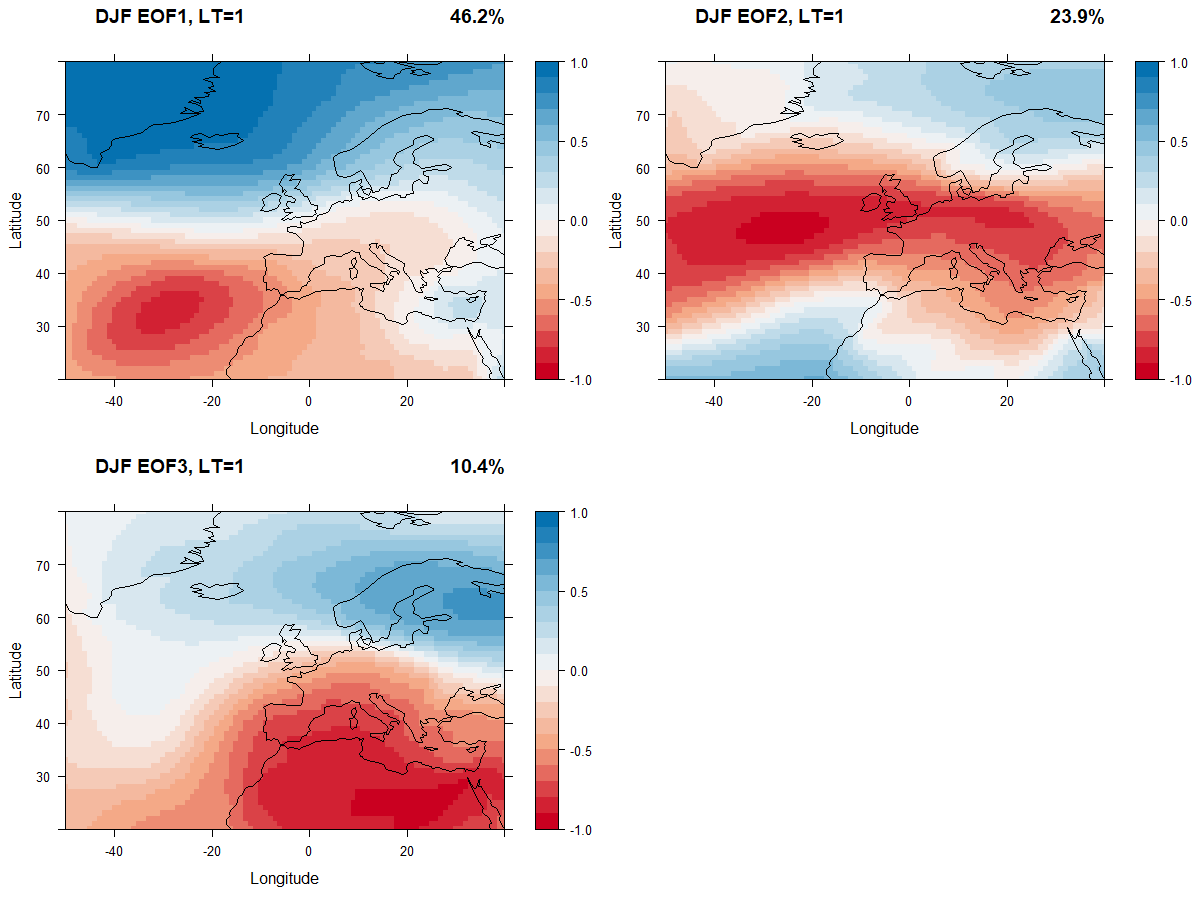


**Figure S4 First three EOFs of winter MSLP from GloSea5 at LT1**


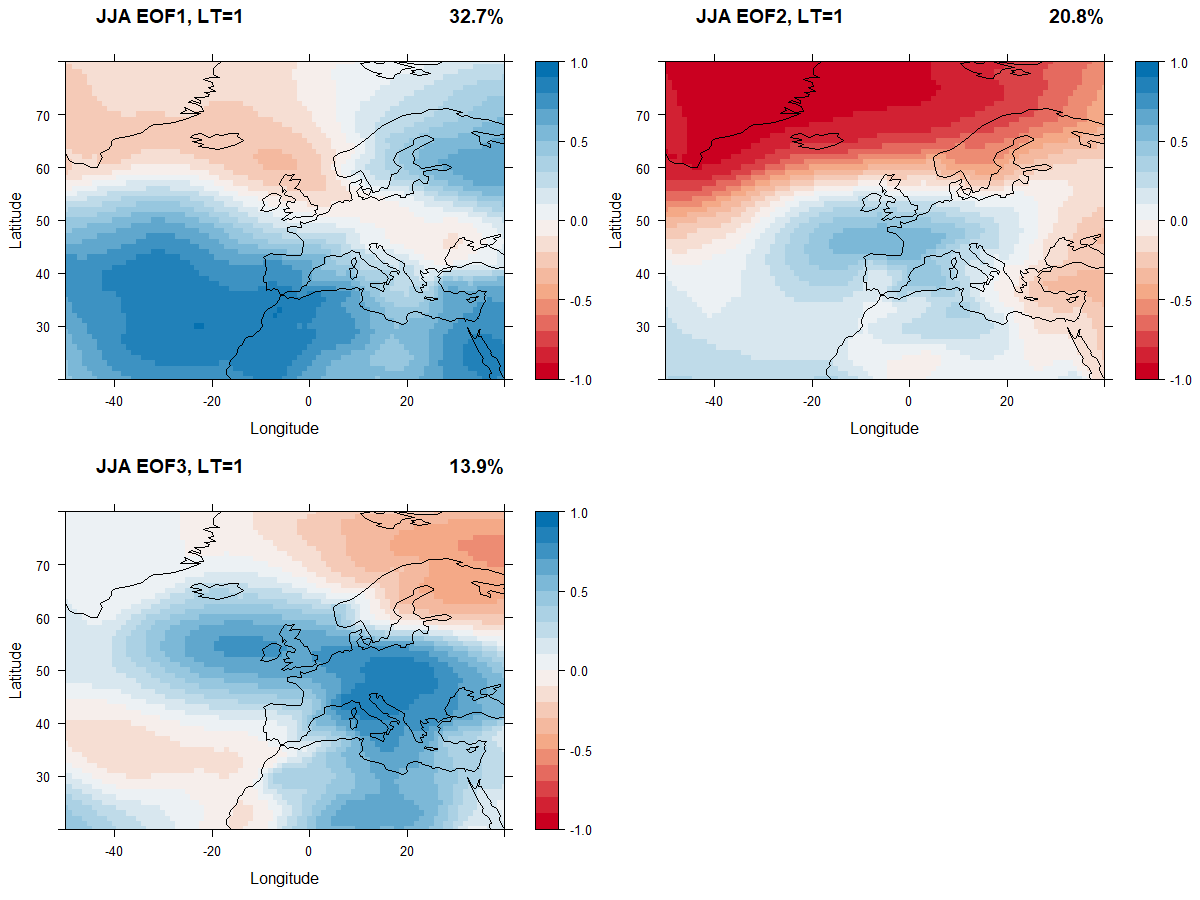


**Figure S5 As in Fig.S4 but for summer**


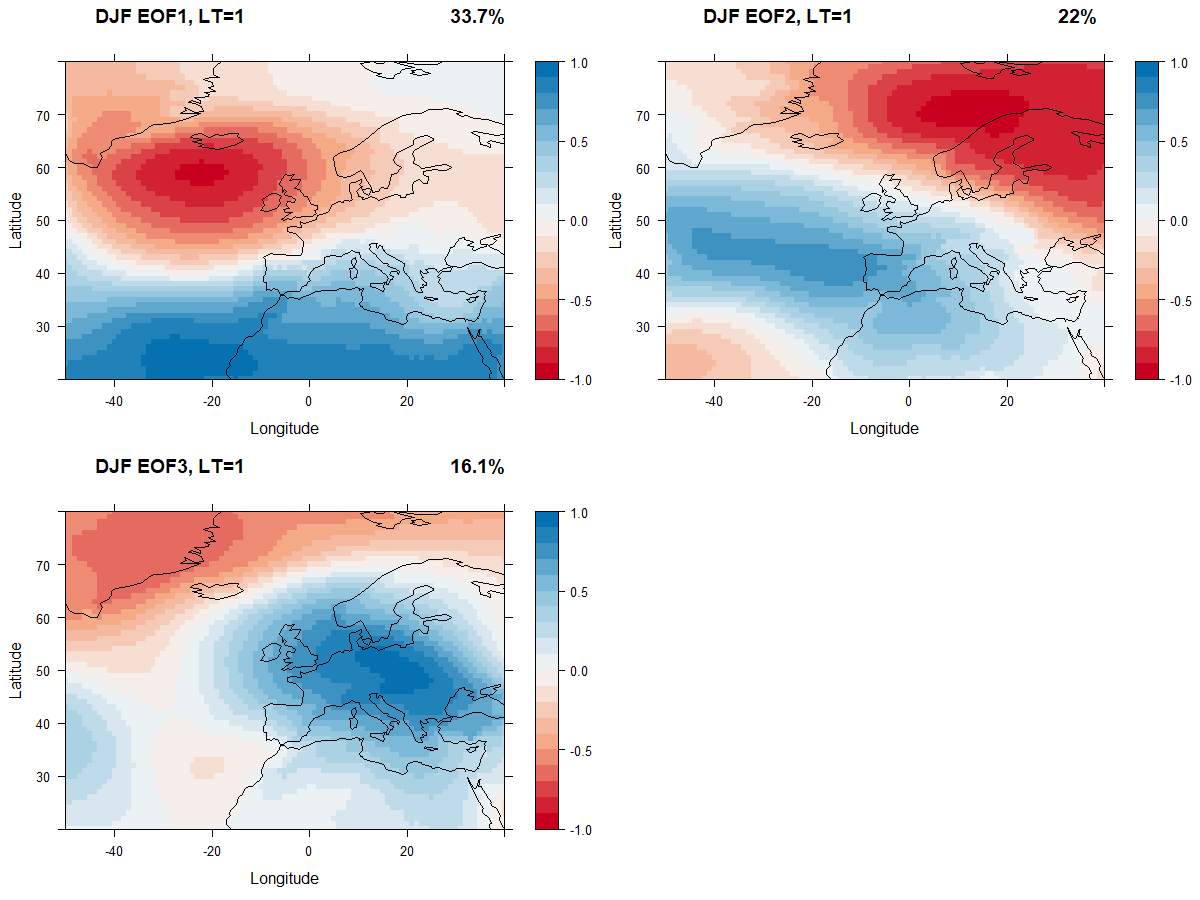


**Figure S6 First three EOFs of winter MSLP from SEAS5 at LT1**


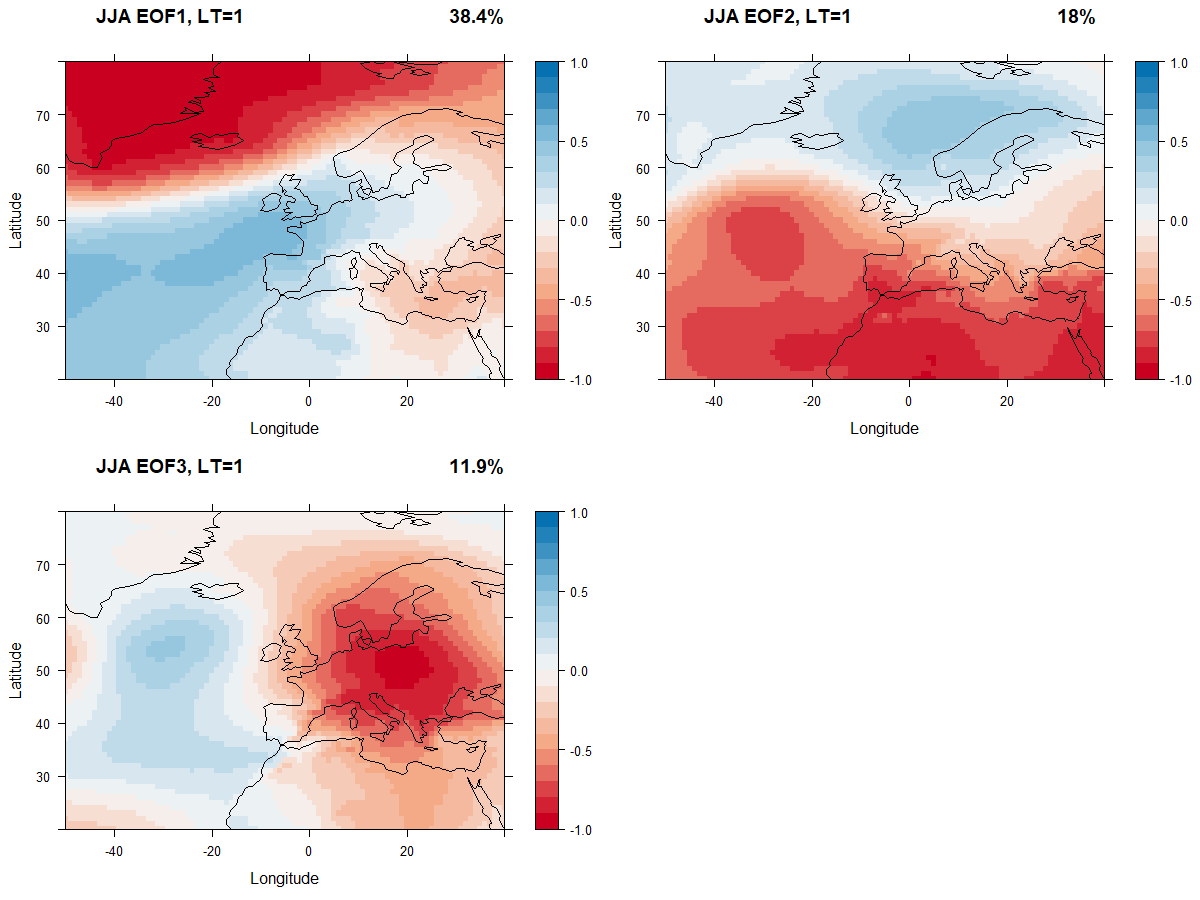


**Figure S7 As in Fig. S6 but for summer**

| 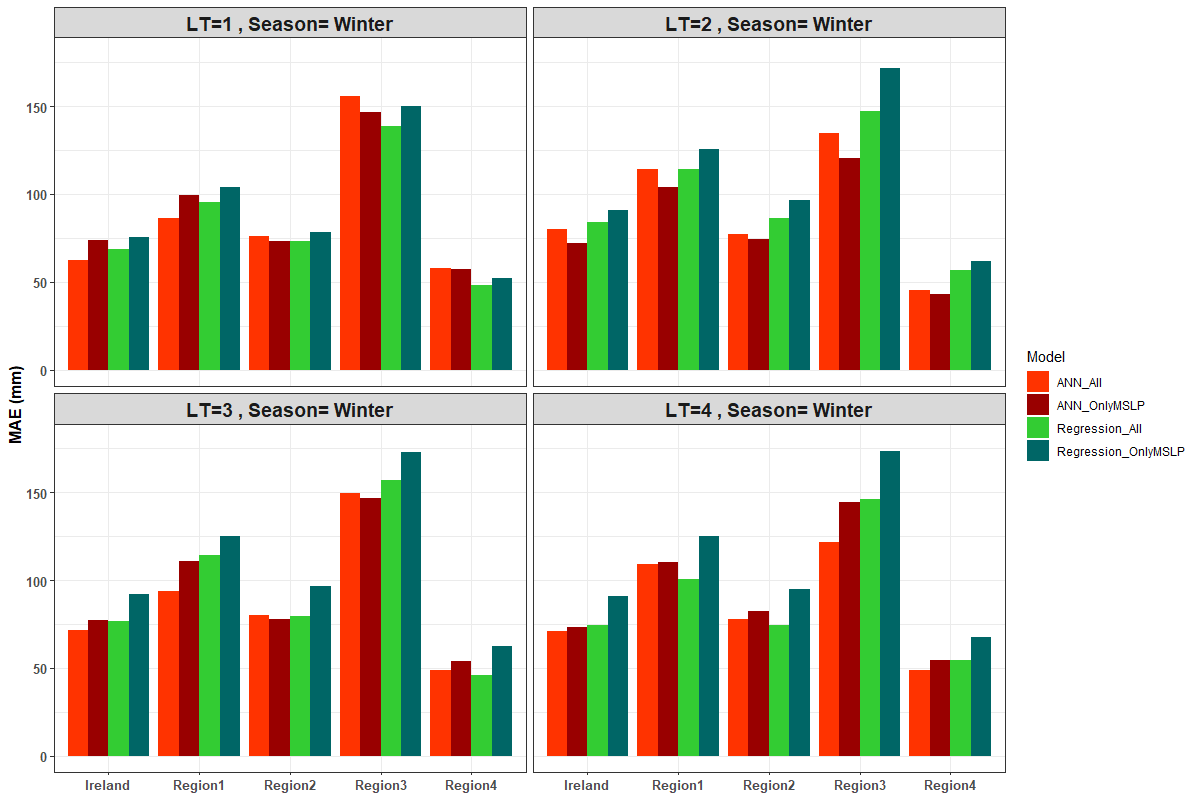 | **(a)** |
| --- | --- |
| 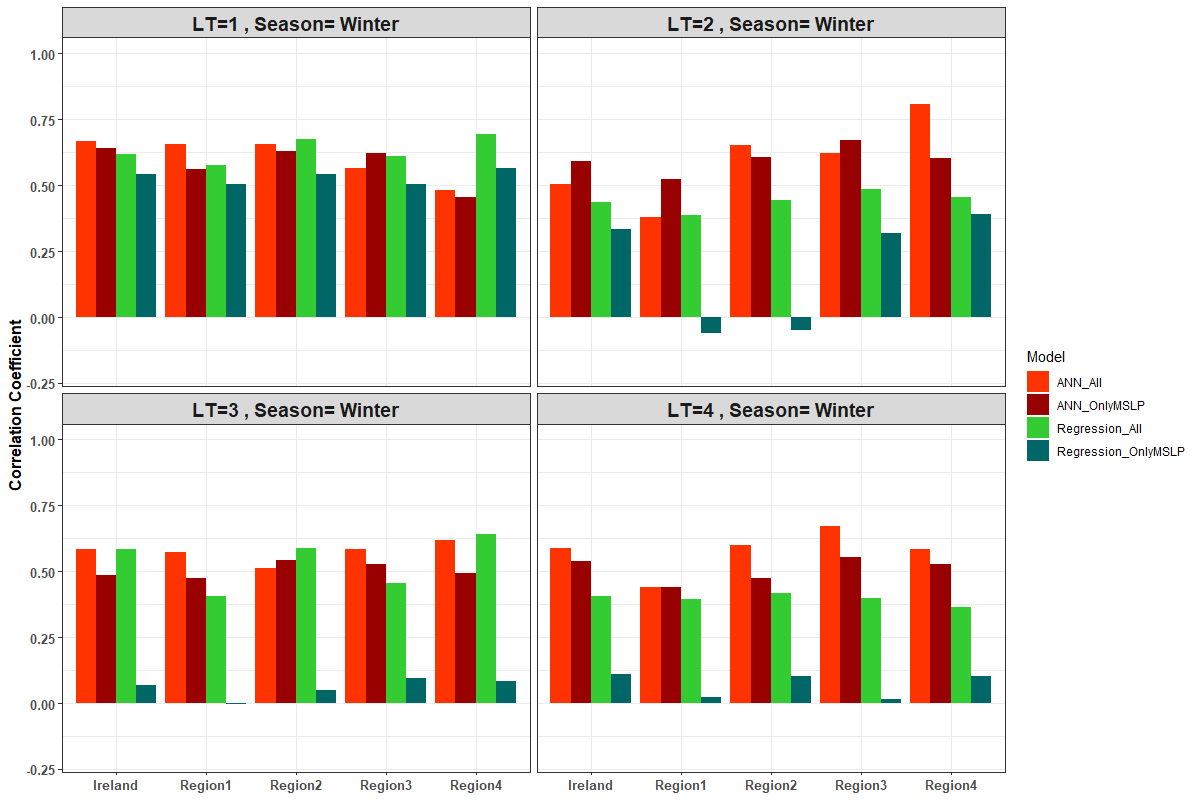 | **(b)** |

**Figure S8 Skill of ANN and MLR models in forecasting winter precipitation using all available predictors or only MSLP-based indices assessed using the a) MAE and b) correlation coefficient**

| 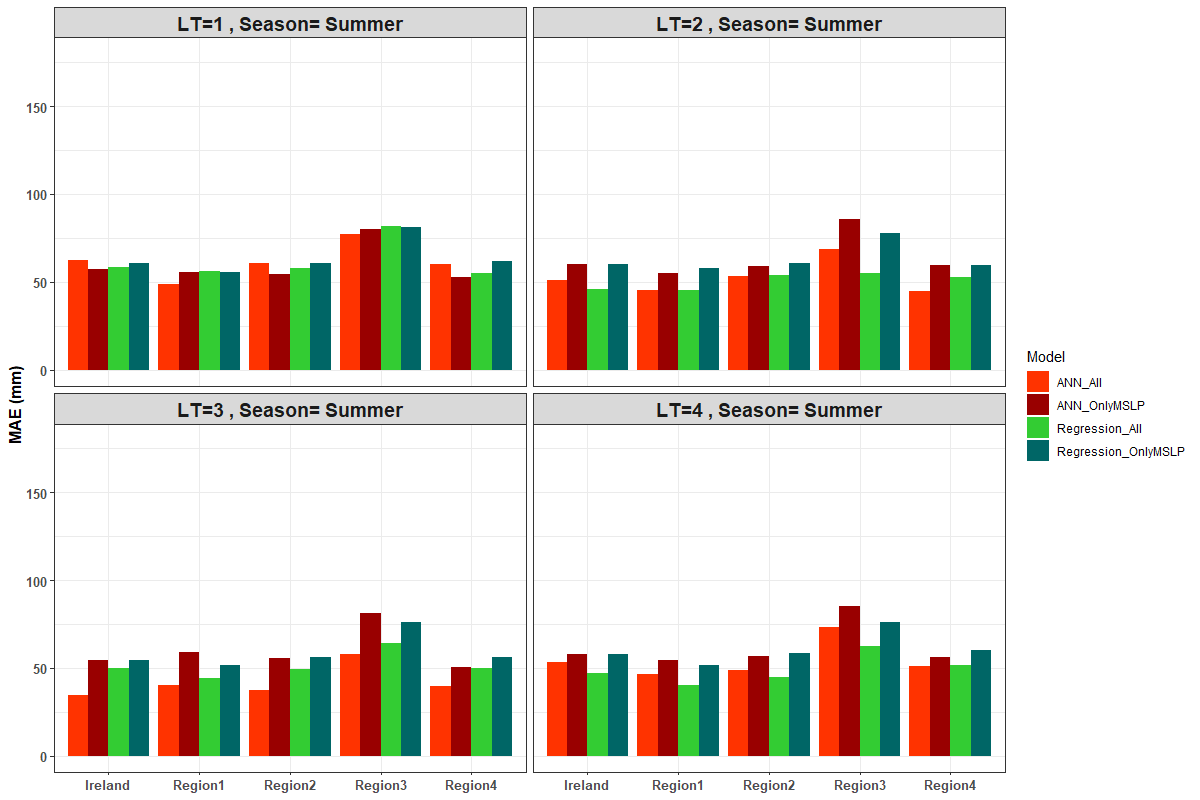 | **(a)** |
| --- | --- |
| 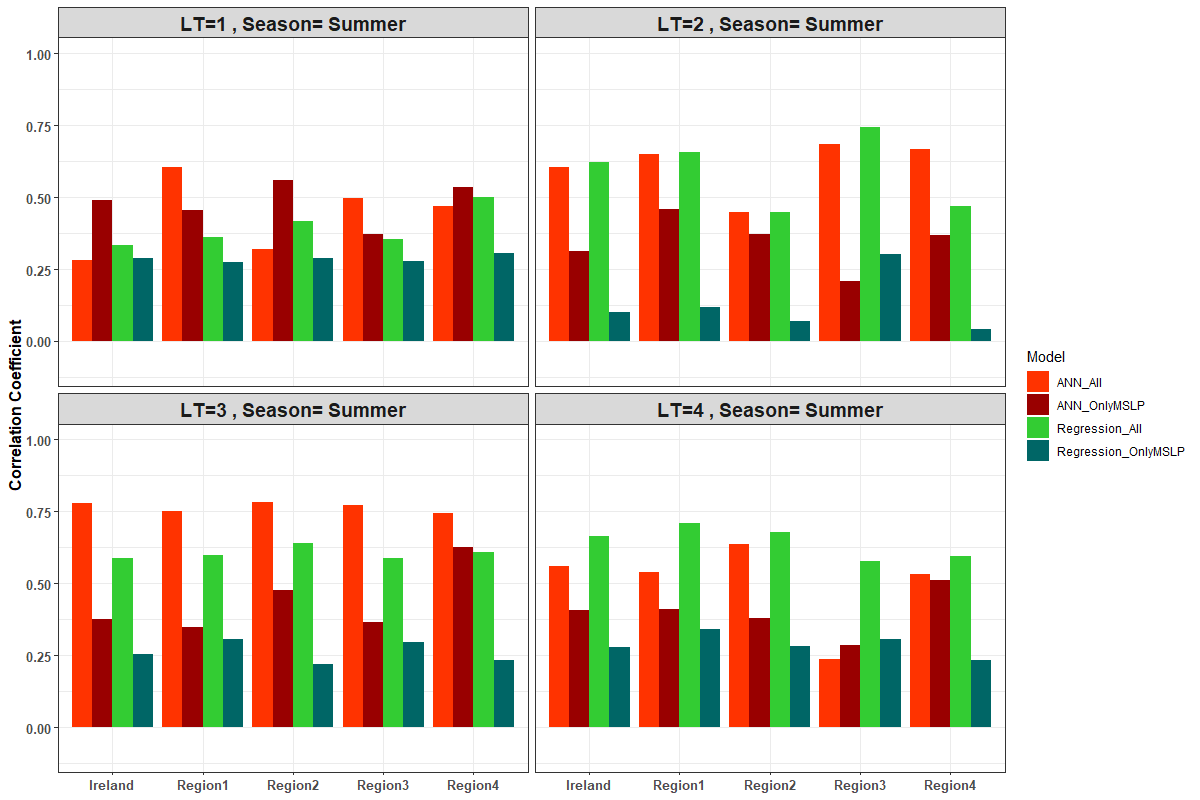 | **(b)** |

**Figure S9 As in Fig. S8, but for summer**

**
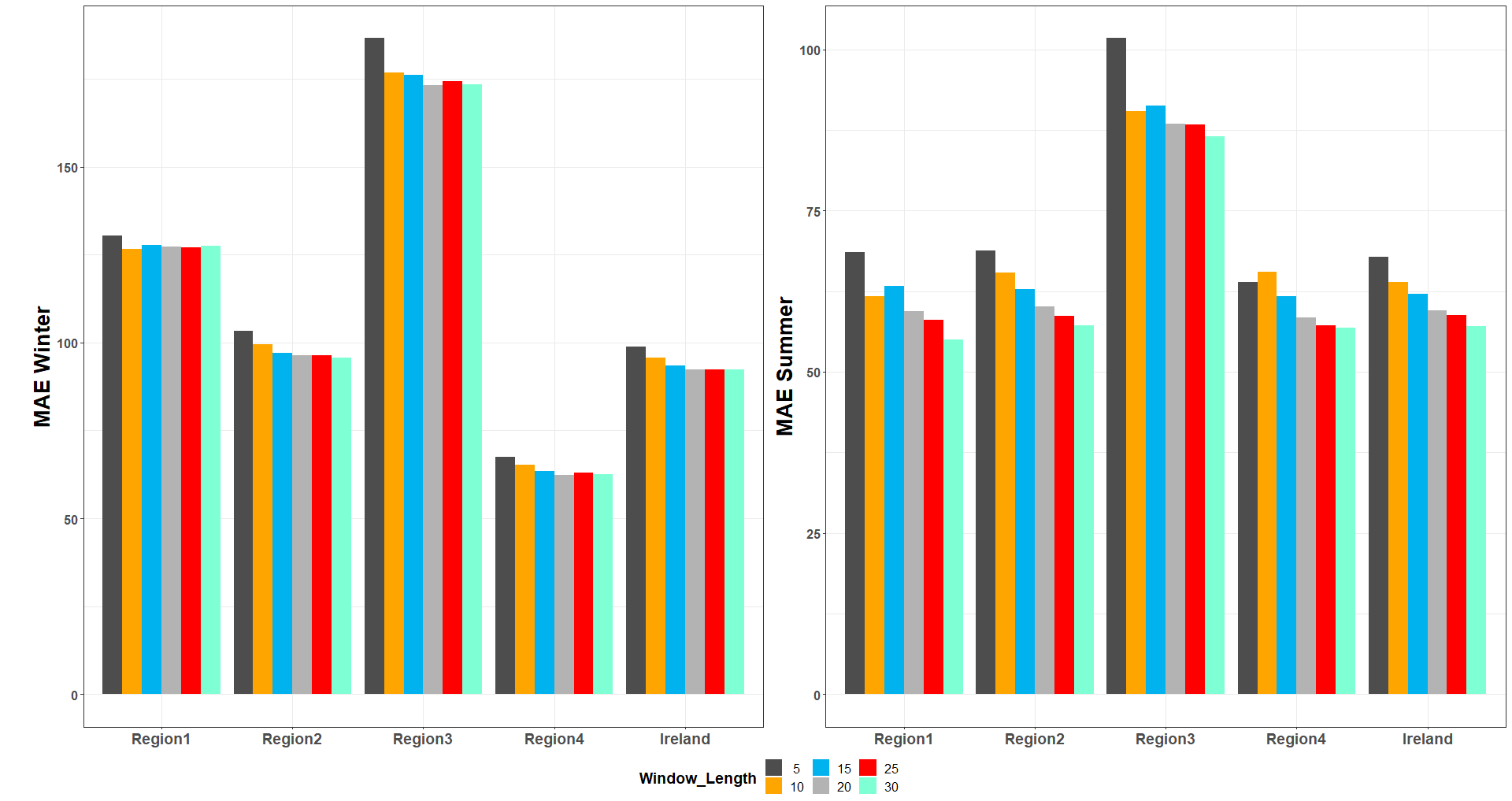
**

**Figure S10- Forecast error (MAE) of climatology depending on window length of the averaging period (years) in a) winter and b) summer**

|  | **(a)** | |
| --- | --- | --- |
|  | **(b)** |  |

**Figure S11- The correlation between observed and predicted precipitation for different lead-times and methods averaged across regions in a) winter and b) summer**

|  |  |
| --- | --- |
|  |  |

**Figure S12- Performance of the ANN and MLR models based on the correlation coefficient for predictors selected based on an exhaustive search method (blue) and fixed predictors (orange) for winter rainfall in various regions across the Island of Ireland.**
